# Systems analysis of multiple regulator perturbations allows discovery of virulence factors in *Salmonella*

**DOI:** 10.1186/1752-0509-5-100

**Published:** 2011-06-28

**Authors:** Hyunjin Yoon, Charles Ansong, Jason E McDermott, Marina Gritsenko, Richard D Smith, Fred Heffron, Joshua N Adkins

**Affiliations:** 1 Department of Molecular Microbiology and Immunology, Oregon Health & Science University, Portland, Oregon 97239, USA; 2 Biological Sciences Division, Pacific Northwest National Laboratory, Richland, WA, 99352, USA

## Abstract

**Background:**

Systemic bacterial infections are highly regulated and complex processes that are orchestrated by numerous virulence factors. Genes that are coordinately controlled by the set of regulators required for systemic infection are potentially required for pathogenicity.

**Results:**

In this study we present a systems biology approach in which sample-matched multi-omic measurements of fourteen virulence-essential regulator mutants were coupled with computational network analysis to efficiently identify *Salmonella *virulence factors. Immunoblot experiments verified network-predicted virulence factors and a subset was determined to be secreted into the host cytoplasm, suggesting that they are virulence factors directly interacting with host cellular components. Two of these, SrfN and PagK2, were required for full mouse virulence and were shown to be translocated independent of either of the type III secretion systems in *Salmonella *or the type III injectisome-related flagellar mechanism.

**Conclusions:**

Integrating multi-omic datasets from *Salmonella *mutants lacking virulence regulators not only identified novel virulence factors but also defined a new class of translocated effectors involved in pathogenesis. The success of this strategy at discovery of known and novel virulence factors suggests that the approach may have applicability for other bacterial pathogens.

## Background

The interactions between intracellular pathogen and host can be complex involving sophisticated offensive and defensive strategies by both organisms. Developing a systems level understanding of the virulence program of a pathogen, both in terms of the regulatory pathways and the virulence-related proteins that execute this program is important to effectively combat persistent and adapting pathogens [1-3]. Combining high-throughput characterization of proteins and gene transcripts under multiple different conditions relevant to virulence provides a wealth of information that can be mined to provide useful leads for further investigation or used as the basis of predictive models.

*Salmonella enterica *serovar Typhimurium (STM) is a facultative intracellular bacterial pathogen with a broad host range capable of infecting birds, reptiles, mice, humans and other mammals. In humans, it is a leading causative agent of gastroenteritis with significant impacts on childhood mortality in the developing world [4] and among HIV positive patients in Sub-Saharan Africa [4,5]. In susceptible mice, STM causes a lethal systemic infection. Because its symptoms resemble human typhoid fever caused by the *S. enterica *serovar Typhi, which only infects man, STM-mediated systemic infection in mice represents an established model system to investigate the pathogenesis and immunology of typhoid fever in humans [6,7]. STM is an intracellular pathogen that can replicate in a variety of cell types, but is most frequently found in monocytes and neutrophils following infection where it is located within a specialized host-membrane bound body, the *Salmonella*-containing vacuole (SCV) [8,9]. STM is able to avoid lysosomal fusion with the SCV and thus evade destruction [10,11].

During systemic infection a sophisticated regulatory network processes and integrates a variety of hostile environmental cues including acidic pH [12], antimicrobial peptides [13] and reactive oxygen species [14] within macrophages triggering induction of specific subsets of genes to adapt to the growth environment, evade the innate immune response, and prevent lysosomal fusion. The coordinated action by this regulatory network involves tight regulation of numerous virulence-related factors including the *Salmonella *Pathogenicity Island-2 (SPI-2) type III secretion system (T3SS) [15] and secreted proteins. SPI-2, located at 31 minutes on the chromosome, encodes the structural components of SPI-2 T3SS as well as regulators and secretion effectors. SPI-2 T3SS delivers a variety of effector proteins into host cells to manipulate host cellular activities. Functions of SPI-2 effector proteins are diverse, including blocking phago-lysosome fusion, subverting inflammatory response, and modulating motility of infected cells [15].

The identification of genes that are required for STM virulence using high-throughput "omics" technologies is an area of considerable experimental investigation [16-20]. The general approach employed in most of these studies involves profiling STM mRNA or protein abundances following infection of host cells or under infection-mimicking conditions versus standard laboratory conditions, with pathogen genes exhibiting higher mRNA or protein abundance under infectious conditions identified as potential virulence factors. However a challenge with this approach is deconvoluting virulence-relevant and -irrelevant effects. Omics methods applied to identify virulence factors frequently identify gene products related to cellular stresses and basal metabolism, which are common responses to growth in a hostile host environment but may not be directly involved in virulence. In order to weed out virulence-irrelevant responses, expression profiles of isogenic avirulent strains lacking essential virulence regulators [21,22] were compared in parallel. When considered individually, any strain missing a virulence regulator may show alterations in a complex regulatory network that includes both virulence factors and genes necessary to respond to specific environmental conditions encountered by free living bacteria as well. Therefore, evaluating commonly converging effects of multiple virulence regulators and in the context of the global regulatory network during infection or, at the very least, under conditions that mimic the host environment represents an attractive novel strategy for the identification of proteins that are required for STM virulence.

In this study, we exploited multiple omics datasets (transcriptomic and proteomic) across multiple regulatory deletions under selected environmental conditions to determine a co-expression network that contains information about regulatory interactions for the infectious environment, which necessitated integration of the omics-derived data utilizing computer-aided network analysis facilitated by the context likelihood of relatedness (CLR) algorithm [23]. Using this approach we identified previously unrecognized virulence factors as well as well-characterized virulence factors that were co-regulated by essential virulence regulators in a coordinated manner. Excitingly, the novel virulence proteins discovered through this approach were categorized into a new class of virulence effectors, which were translocated into the host cytoplasm independently of *Salmonella *type III secretion mechanisms.

## Results

### Rationale for strains used and study design

To date, more than 300 genes have been annotated as regulatory genes in *Salmonella *and of those tested (83) we showed that 14 regulators including SpvR, FruR, IHF, PhoP/PhoQ, SsrA/SsrB, SlyA, Hnr, RpoE, SmpB, CsrA, RpoS, CRP, OmpR/EnvZ, and Hfq are required for virulence regulation during systemic infection in an acute mouse infection model [24]. *Salmonella *strains lacking these regulators were not only attenuated for mouse virulence, but also exhibited dysregulation of many known *Salmonella *virulence factors, including SPI-2 genes. These regulators were linked to each other through positive or negative feedback regulation and activated SPI-2 genes in a coordinated manner [24]. Thus we reasoned that genes that are coordinately controlled by this set of 14 virulence regulators and co-regulated with known virulence factors are likely to be important for *Salmonella *pathogenesis. We employed large-scale sample-matched multi-omic measurements of *Salmonella *strains lacking these regulators, coupled with computational network analysis to efficiently identify such genes, which represent conservatively selected putative virulence factors.

### Sample-matched multi-omic profiling to infer putative virulence factors

Recent studies have discovered that a large number of *Salmonella *genes, estimated to be at least 20% of all genes, are post-transcriptionally regulated by a single regulatory mechanism [25,26]. Thus mRNA and protein measurements from each regulatory mutant and wild-type strain were used as complementary methods to characterize the global/genome-wide effects for each regulator. Because some of the *Salmonella *regulatory mutant strains (e.g. Δ*himD*, Δ*rpoE*, and Δ*hfq*) survived so poorly within macrophages that preparing mRNA or protein from intracellular bacteria was not readily practical, we have used *in vitro *growth conditions that duplicate many aspects intracellular conditions, as previously described [24,26,27]. *Salmonella *strains were grown independently in triplicate in four *in vitro *conditions: Luria-Bertani (LB) logarithmic and LB stationary phases, and two acidic minimal medium (AMM) conditions, AMM1 and AMM2 [24,26]. LB broth, given its high osmolarity and nutrient-rich condition, partially reproduces the small intestine lumen environment, while the AMM conditions, providing a low pH, low magnesium, and nutrient-deficient condition, partially mimic the intracellular milieu within the *Salmonella*-containing vacuole (SCV) [28,29].

Changes in gene expression and protein abundance between the parent wild-type strain and each isogenic mutant were evaluated across the four growth conditions. Transcriptomic analysis quantified 4517 genes across 59 profiling datasets [24]; mass spectrometry-based label free proteomics combined with the AMT tag approach [30], quantified a total of 1349 proteins (Additional file 1, Table S1) across 63 profiling datasets. The large volume of data generated (over 300,000 data points from the combined omics analyses) necessitated a computational approach to integrate the data into a single systems-level view that allows efficient and systematic identification of biomolecules important for *Salmonella *pathogenesis.

### Computational analysis of an integrated multi-omics network reveals putative virulence factors

To identify proteins important for *Salmonella *pathogenesis (i.e. virulence factors) in an efficient and systematic fashion we employed the context likelihood of relatedness (CLR) algorithm [23]. The CLR algorithm can infer regulatory networks from transcriptomic data for experimentally verified regulatory interactions [23]; and the topology of a gene in the inferred network is correlated with phenotypic properties of the gene, for example, its role in virulence [31]. We have previously inferred and validated a regulatory network from the transcriptomic profiles of the described regulatory mutants using CLR [24]. In the current study we applied CLR to the proteomics data to infer protein association networks. These networks contain information about the general regulatory structure of *Salmonella *at the protein abundance level, but are not explicit regulatory networks. This resulted in a network of proteins and general associations between them that might indicate regulation (as in inferred transcriptional networks [24]), complex formation, or pathway membership.

**Figure 1:**
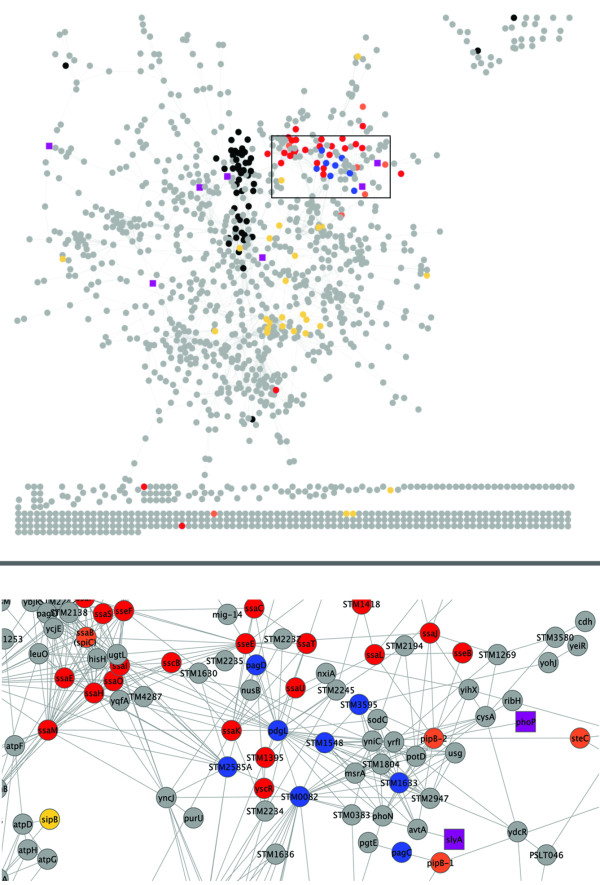
**Association network inferred from integrated proteomic and transcriptomic data**. The CLR method was used to infer association relationships between proteins on the basis of their abundance profiles (Z score > 6.0). The resulting network was extended by combining with association relationships inferred from transcriptomics data (Z score > 8.0). The network was visualized in Cytoscape. The nodes in the network are colored as follows: black, ribosomal proteins; red, SPI-2 proteins; yellow, SPI-1 proteins; orange, effectors which are secreted through SPI-2 T3SS but not in SPI-2; purple, regulators known to be essential for virulence; blue, targets identified in this study.

In order to identify novel virulence-related proteins for further investigation we chose to examine proteins with similar abundance profiles to SPI-2 proteins over all conditions and mutants. To include high-confidence relationships with gene products not observed in the proteomics network, we combined the protein association network with a transcript association network inferred from the corresponding transcriptomics data. Extending our previous work analyzing networks inferred from transcriptomics [31], we discovered that integrating data in this way increased our ability to identify genes/proteins essential for virulence using network topology, over either of the individual networks (see Additional file 2, Methods S1; Additional file 3, Figure S1; Additional file 4, Table S2). We then combined the proteomic and transcriptomic data under stringent thresholds: high confidence protein to protein (Z score > 6.0) and gene to gene (Z score > 8.0) relationships were included. The resulting network and the region containing the majority of the SPI-2 proteins are shown in Figure 1. Clusters were identified from the network using hierarchical clustering. We examined different clustering thresholds and chose the threshold for which the SPI-2 effector proteins were all contained in one cluster of minimal size (Additional file 5, Table S3, cluster 1). The cluster containing the SPI-2 genes, corresponding to the region of the network is highlighted in Figure 1. A number of the genes/proteins in this cluster were also identified as highly central in the topological network analysis ( Additional file 2, Methods S1 and Additional file 4, Table S2) and essential virulence genes were significantly enriched in this cluster (p-value < 0.01) confirming its importance in virulence. In order to verify the association of this cluster with *Salmonella *virulence, a subset of novel genes/proteins within this cluster was selected including STM0082 (*srfN*/SrfN), STM1244 (*pagD*/PagD), STM1246 (*pagC*/PagC), STM1548, STM1599 (*pdgL*/PdgL), STM1633, STM2585A and STM3595, and their role in virulence was investigated as described below. Analysis of these proteins showed that they are coordinately regulated at the protein abundance level by HimD, PhoP/PhoQ, SsrA/SsrB, RpoE, RpoS, CsrA, and OmpR/EnvZ, (Additional file 6, Figure S2) consistent with our previously published results showing that virulence genes were coordinately regulated [24]. Three of these candidate proteins were identified as bottlenecks in our topological analysis, and thus are postulated to mediate important transitions in the system; SrfN, PdgL, and STM2585A.

### Validation of computational predictions

**Figure 2:**
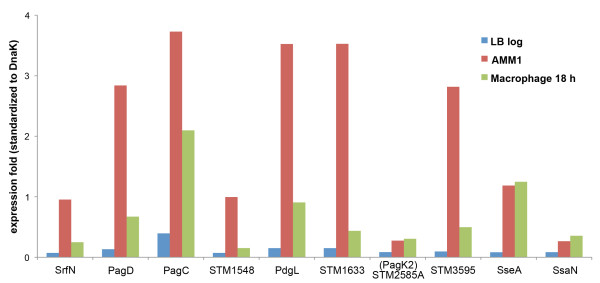
**Expression of putative effector proteins under *in vitro *and *ex vivo *conditions**. Candidate proteins and two SPI-2 proteins (SseA and SsaN) were labeled with 2HA or CyaA' and their expression was examined under ***in vitro ***(LB log phase and AMM1) and ***ex vivo ***(RAW264.7 cells infected with HA or CyaA'-tagged strains) conditions. In order to compare protein expressions across conditions, the signal intensity in Western blot analyses (Additional file 7, Figure S3) was quantified via ImageJ software based analysis (http://rsb.info.nih.gov/ij/). Protein levels in LB log phase, AMM1, and macrophages (18 h post-infection) conditions were normalized using DnaK levels and the expression fold was compared in three conditions.

Computational analysis of the integrated multi-omic datasets predicted a set of putative virulence proteins. To validate our network predictions, genes encoding the eight candidate proteins were chromosomally tagged with the hemagglutinin (HA) epitope or the amino-terminal domain of *B. pertussis *adenylate cyclase (CyaA'), and their protein abundance profiles were examined in each of the four *in vitro *conditions (Additional file 7, Figure S3A). All eight proteins (SrfN, STM1548, PdgL, STM1633, STM3595, PagC, PagD, and STM2585A) had increased expression under acidic minimal media (AMMs), which partially mimics the host intracellular environment, compared to log phase growth in LB media. We next investigated expression of the eight proteins in a more physiologically relevant environment, within host macrophage cells. RAW264.7 murine macrophages were infected with the HA or CyaA'-tagged *Salmonella *strains, and the expression of tagged-proteins was examined at 6 and 18 hours post-infection (Additional file 7, Figure S3B). Although expression levels varied between the *Salmonella *strains, all eight proteins were induced more strongly inside macrophages than in LB log phase condition (Figure 2), which is a typical characteristic of well-characterized SPI-2 virulence proteins/factors [32]. Taken together, our immunoblot experiments validate the network prediction, showing that the putative virulence factors inferred from the regulatory network had a similar expression profile to that of SPI-2 components and suggest that SrfN, STM1548, PdgL, STM1633, STM3595, PagC, PagD, and STM2585A may be important for intracellular growth.

### SrfN, PagC, PagD, and STM2585A are translocated into the host cytosol

**Figure 3:**
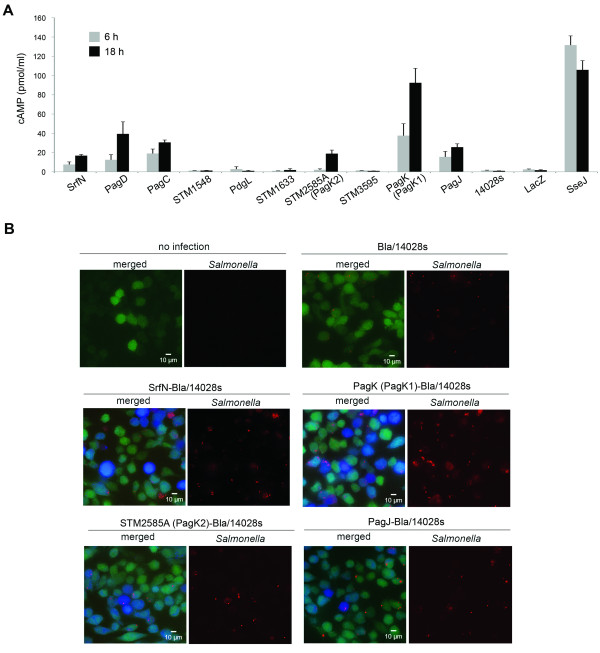
**Translocation of SrfN and Pag proteins into the macrophage cytosol**. **A**. RAW264.7 cells were infected with *Salmonella *expressing CyaA'-tagged proteins and intracellular cAMP was measured at 6 and 18 h post-infection. Wild-type *Salmonella *(14028s, not expressing CyaA'), 14028s transformed with pMJW1791 [41] expressing β-gal-CyaA' (LacZ, an intracellular protein), and 14028s expressing chromosomal SseJ-CyaA' (SseJ, a well-known SPI-2 effector) were used in infection as controls. **B**. SrfN and PagK (PagK1)/STM2585A (PagK2)/PagJ were labeled with β-lactamase and RAW264.7 cells were infected with wild-type *Salmonella *and Bla fusion strains for 18 hours. Cells were loaded with CCF4-AM for 2 hours. CCF4-AM cleaved by translocated Bla-tagged proteins changed emission wavelength from 528 nm (green) to 457 nm (blue). *Salmonella *strains were transformed with pWKS30-Tomato and shown in red. pWKS30-Tomato encodes an episomal Bla lacking a secretion signal and wild-type *Salmonella *transformed with pWKS30-Tomato was used as a negative control. As a positive control, SseJ, an effector translocated via SPI-2 T3SS, was tagged with Bla and its translocation is shown in Additional file 12, Figure S8.

An important feature of *Salmonella *pathogenesis is the active translocation of effector proteins into the host cell cytoplasm to create a replicative niche and elude the host immune response. While more than 40 such secreted virulence factors have been reported to date, the full repertoire of effectors has not been catalogued [33,34]. Therefore, we determined whether the eight putative virulence factors were translocated to the macrophage cytoplasm. SrfN, STM1548, PdgL, STM1633, STM3595, PagC, PagD, and STM2585A were each fused with CyaA', a potent cyclase that requires calmodulin for synthesis of cAMP from ATP [35], and intracellular cAMP levels were measured at 6 and 18 hours post-infection of macrophages (Figure 3A). Calmodulin is ubiquitous within animal cells but not within *Salmonella *or the SCV, the main *Salmonella *reservoir within host cells [11]. STM0082, which was recently identified as SrfN [27], was secreted into the macrophage cytosol, although the secretion level was low compared to that of SseJ, a well-studied effector protein secreted through SPI-2 T3SS [36]. Besides SrfN, three Pag proteins including PagD, PagC, and STM2585A were translocated into the host cytoplasm. STM2585A, encoding Gifsy-1 prophage protein, has high sequence homology with PagK (95%) and PagJ (83%) (Additional file 8, Figure S4). Therefore, PagK and PagJ were tagged with CyaA' and their translocation was examined in macrophages in parallel. Although the expression levels (Additional file 7, Figure S3B) and translocation levels (Figure 3A) were variable between these, all three PagK homologues were expressed inside host cells and translocated into the cytosol. PagK, PagJ, and STM2585A are small proteins composed of 66, 66, and 75 amino acids (aa), respectively. Failure to identify PagK and PagJ via global transcriptomics and proteomics may be a consequence of the limited number of possible tryptic peptides available for protein identification via SEQUEST, relatively low abundance without sub-cellular enrichment, and misidentification among PagK homologues due to their near sequence identity. Translocation of SrfN, PagK, PagJ, and STM2585A was further validated using CCF4-AM cleavage (Figure 3B and Additional file 9, Figure S5). The four putative effector proteins were fused with ß-lactamase (Bla), which can cleave CCF4-AM to change its emission spectrum from green to blue when it is translocated into the cytoplasm. When macrophages were loaded with membrane-permeable CCF4-AM, cells infected with *Salmonella *Bla-fusion strains exhibited blue fluorescence (Figure 3B), which is consistent with the previous cAMP assay results. From our results, we infer that SrfN and Pag proteins are potential virulence effectors translocated into the host to manipulate cellular functions during *Salmonella *systemic infection.

### Virulence properties of SrfN and PagK homologues

**Figure 4:**
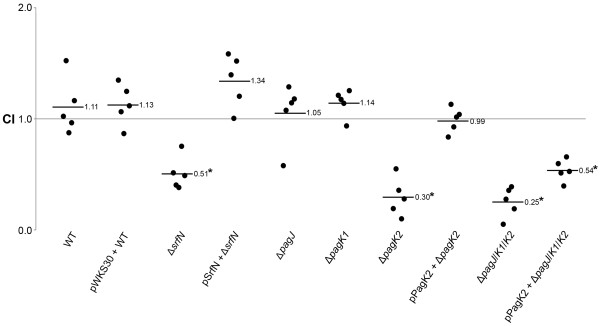
**Virulence study on putative effector proteins**. To compare the viability of deletion strains lacking putative effector proteins with that of wild-type *Salmonella*, competitive infection was performed in five 129SvJ mice for each mutant. Δ*srfN*, Δ*pagK2*, and Δ*pagJ*/*pagK1*/*pagK2 *were transformed with pSrfN or pPagK2 to test virulence complementation, whereas wild-type *Salmonella *was transformed with an empty plasmid, pWKS30. A reference strain (MA6054) harboring arabinose-inducible β-galactosidase activity and each test (wild-type or mutant) strain were mixed equivalently and 10,000 CFU of the mixed cells was used to infect a mouse. Mice were sacrificed at 7 days post-infection and the spleen lysates were spread on plates containing X-gal and arabinose to compare the numbers between the wild-type strain and the mutant strain. The competitive index (CI) was determined by the formula described in Methods. Each circle represents the CI for a single mouse in each group. A CI of less than 1 indicates that a test strain is outcompeted by the reference strain in mouse. The averaged CI for each group is shown as a solid line and statistically significant p-value is indicated with an asterisk (p-value < 0.001, Student's t-test).

The fact that SrfN and several Pag proteins including PagC, PagD, and three PagK homologues were expressed inside macrophage cells and were translocated into the host cytoplasm prompted us to investigate their roles in *Salmonella *virulence. These proteins might contribute to *Salmonella *survival following translocation. SrfN was demonstrated to play a role in *Salmonella *fitness within the host [27]. However, deletions of *pagC, pagD*, and *pagK *were reported to have no effect on virulence [37-40]. Gunn *et al*. identified a homologue of *pagK, pagJ*, in a 1.6 kb duplicated DNA region present in ATCC 14028 (but not LT2), but did not observe virulence attenuation with deletions of these homologous genes individually or in combination [39]. STM2585A, which was identified as another PagK homologue in this study, was expressed and translocated simultaneously with PagK and PagJ. Therefore, we presumed it was possible that the three homologues might carry out a redundant function and that there would be no phenotype unless all three were deleted. STM2585A was designated as PagK2 while PagK was renamed PagK1 to distinguish them. In-frame deletions of *srfN, pagK1, pagK2*, and *pagJ *were constructed individually or in combination and their effects on bacterial proliferation ability were investigated in mice that were co-infected with an equivalent mixture of the wild-type and each deletion mutant strain to evaluate competitive index (CI) (Figure 4). When the fitness of deletion strains were compared with that of wild-type bacteria in the same host, three strains, Δ*srfN*, Δ*pagK2*, and Δ*pagJ*/*pagK1*/*pagK2*, were out-competed by wild-type bacteria resulting in a CI value less than 1. The growth defect of Δ*srfN *and Δ*pagK2 *was complemented *in trans *by a plasmid expressing SrfN and PagK2 respectively (Figure 4). The replication of *Salmonella *lacking PagJ or PagK1 only was equivalent to that of wild-type bacteria, raising the possibility that the attenuated survival of the triple deletion strain (*pagJ*/*pagK1*/*pagK2*) was likely attributable to the loss of PagK2. However, the attenuated fitness of the triple deletion strain was restored only in part by *in trans *PagK2 expression, suggesting at least partial functional redundancy among three PagK-related proteins (Figure 4). Modest attenuation of *Salmonella *mutants lacking effector proteins, observed in the virulence tests, may be due to functional redundancy between virulence factors as previously described [41,42]. The effect of PagC and PagD on *Salmonella *virulence was examined through mice infection as well, however they did not affect *Salmonella *fitness in mice (unpublished data) as reported previously [38,39] and they were excluded from further investigation which aimed to identify new virulence effectors.

### Exploring the translcoation pathway for SrfN, PagJ, PagK1, and PagK2

**Figure 5:**
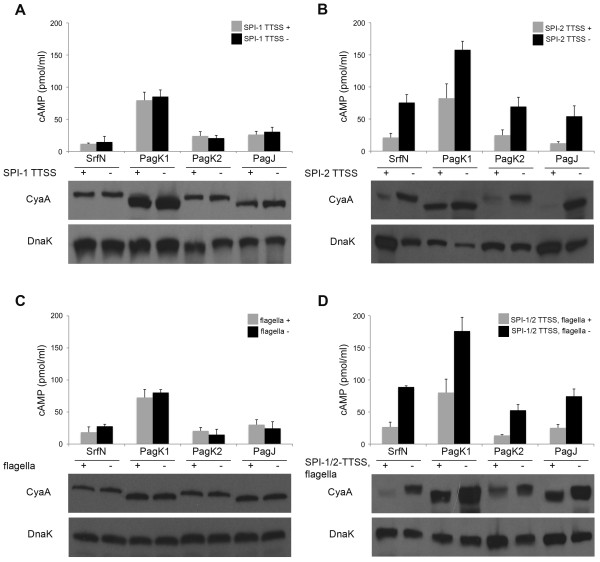
**Translocation of SrfN and PagK1/PagK2/PagJ independent of SPI-1/2 T3SSs and flagella system**. Genes of *invA, ssaK*, and *flgB *were deleted individually or in combination to block SPI-1 T3SS (A), SPI-2 T3SS (B), flagella system (C), or all three secretion machineries (D), respectively in *Salmonella *strains expressing CyaA'-tagged SrfN, or PagK1/PagK2/PagJ. Macrophage cells infected with *Salmonella *were lysed at 18 h post-infection to examine translocation and expression levels of SrfN and PagJ/K1/K2. Translocation of CyaA' fusions into the macrophage cytosol was measured using cAMP assay (top graphic figures) and the intracellular expression levels of CyaA'-tagged proteins were compared in parallel using Western blot analysis (bottom gel figures). DnaK levels were measured to normalize protein amounts loaded between lanes in Western blotting. *Salmonella *without SPI-2 T3SS was attenuated in intracellular growth and exhibited low DnaK levels compared with wild-type *Salmonella*.

The observation that SrfN and PagK homologues, which are tranlocated into host cells, influenced *Salmonella *virulence raised a question of how these effector proteins are translocated. Considering that these proteins exhibited a similar expression profile with SPI-2 T3SS components across 4 different conditions in transcriptomic and proteomic analyses, they were likely to be secreted via SPI-2 T3SS. The T3SS is a specialized needle-like organelle made up of more than 20 components and delivers more than 30 effectors into host cells to create a hospitable environment for *Salmonella *proliferation [43,44]. T3SSs are evolutionarily related to the flagellar system and show structural similarity with this apparatus. In fact, the flagellar system has been shown to be an alternative transport machinery for some virulence factors [45,46]. In order to decipher the secretion pathway of SrfN and PagK homologues, SPI-1/SPI-2 T3SSs and the flagellar system were disrupted individually or in combination by deleting essential components of their secretion apparatuses (InvA in SPI-1 T2SS; SsaK in SPI-2 T3SS; FlgB in flagella). The disruption of secretion systems was verified by reduced motility of Δ*flgB *strain on agar plates and blocked translocation of SipA (a SPI-1 T3SS-translocated effector) and SseJ (a SPI-2 T2SS-translocated effector) in Δ*invA *and Δ*ssaK *strains-infected macrophages (Additional file 10, Figure S6). However, translocation of SrfN and PagK homologues was not abolished by any deletions, even when all three secretion systems were absent (SPI-1 T3SS, SPI-2 T3SS, and flagella) (Figure 5 and Additional file 10, Figure S6). Intriguingly, SrfN and PagK homologue proteins all showed increased protein levels and translocation levels when SPI-2 T3SS was inactivated. Single deletion of the SPI-1 T3SS or flagellar system did not appear to impair bacterial survival in macrophages and revealed equivalent expression and translocation levels of SrfN and PagK homologues to those of wild-type as well, implying that the feedback regulation sensing T3SS is restricted to SPI-2 T3SS. To further elucidate the effects of SPI-2 T3SS on the expression of SrfN and PagK homologues, mRNA levels from intracellular bacteria were compared between a wild-type strain- and the SPI-2 T3SS-defective strain (Δ*ssaK*)-infected macrophages (Additional file 11, Figure S7). Transcription of *srfN *and *pagK1/pagK2*/*pagJ *was not affected by the lack of SPI-2 T3SS, indicating that the increases in SrfN and PagK homologues observed occurred as a consequence of post-transcriptional regulation. The result that SrfN, PagK1, PagK2, and PagJ were translocated into the host cytosol independent of any T3SSs and flagellar system suggests that SrfN and a subset of Pag proteins are translocated by an as yet unidentified mechanism.

## Discussion

In this study, we have investigated the global regulatory network required for *Salmonella *virulence under specific *in vitro *(infection-mimicking) conditions by employing regulatory protein perturbations and high throughput sample-matched omics measurements coupled with computational network analysis. This systems-level analysis of the *Salmonella *virulence program inferred 168 proteins which were clustered close to SPI-2 virulence proteins in the regulatory network and thus were likely to be involved in pathogenicity (Additional file 4, Table S2). A set of these predicted virulence candidate proteins were tested to verify the network prediction, including SrfN, PagD, PagC, STM1548, PdgL, STM1633, PagK2 and STM3595. These eight proteins were expressed more highly within macrophages than under LB log phase condition (Figure 2 and Additional file 7, Figure S3), which is characteristic of the SPI-2-encoded virulence proteins and suggests that their functions may be required for intracellular growth. Interestingly, SrfN, PagC, PagD, PagK2 (and its close homologues PagK1 and PagJ) were translocated to the macrophage cytoplasm (Figure 3), suggesting that these six proteins are secreted virulence effectors interacting with host cellular components to promote bacterial proliferation. In fact, SrfN and PagK homologues were required for *Salmonella *systemic infection in mice (Figure 4).

We showed that the integration of transcriptomics and proteomics data at the network level could provide enhanced predictions of components important for virulence. This is a novel way of integrating these two disparate data types, and should be applicable to other systems. Three of the proteins chosen for investigation had very high betweenness measures in the network (SrfN, PdgL, and STM2585A/PagK2; see Additional file 4, Table S2). It is interesting that the two translocated proteins from this later group, SrfN and PagK2, were also shown to have an effect on virulence in mice. Though a limited validation, this supports our hypothesis that the topology of the network can be informative about the importance of genes/proteins. PdgL was not translocated, but possesses a PhoP box as other PhoP-regulated virulence proteins (e.g., MgtC, PagK, VirK, PipD) responding to the intracellular environment [47,48] and its inactivation rendered *Salmonella *hypervirulent in mice infection, suggesting its crucial role concerned with *Salmonella *intracellular fitness [49], a possibility we are currently examining. Our approach, combining phenotype-specific regulatory mutants with computational analysis of resultant multi-omics data, provides a novel and useful method for prediction of virulence-related genes.

Osborne *et al*. [27] recently reported that *srfN*, an ancestral PhoP-regulated gene, acquired an SsrB-regulatory module during *Salmonella's *evolution to a pathogenic bacterium. We also verified SsrB- and PhoP-dependent transcription of *srfN *measuring mRNA levels during growth in AMM1 medium (Additional file 12, Figure S8). However, they did not observe SrfN translocation to the host cytoplasm [27]. The most probable explanation for this apparent discrepancy is due to the different cell types used, i.e., epithelial cells used in that study versus macrophages used in our study. Secretion of effector proteins may be specific to the intracellular environment, which has been reported for SseJ [8]. SseJ, a SPI-2 effector was not translocated into the cytosol of HeLa cells at least at 2 h post-infection [8]. Macrophage-biased expression was also reported for a number of SsrB-regulated SPI-2 genes [50].

PagK was first identified as a PhoP-activated virulence gene, where Tn*phoA *insertion decreased *Salmonella *virulence significantly in mice [37]. However, a deletion strain lacking *pagK *exhibited a wild-type phenotype in virulence [39]. Although Gunn *et al*. identified a homologue, *pagJ*, in a 1.6 kb duplicated DNA region; they could not reproduce virulence attenuation with deletions of these homologue genes individually or in combination, suggesting that the original transposon insertion may have affected expression of additional genes [39]. This virulence trait is explained by the results described here because the presence of a third related gene was not detected in that study and all three must be deleted to see the largest virulence defect. PagK (or PagK1) has a high amino acid identity with PagK2 (95%) as well as PagJ (83%). Furthermore, all of these three homologues were translocated into the host cytoplasm and negatively regulated in a similar way by SPI-2 type III secretion, suggesting a close correlation and shared properties among three PagK-homologous proteins. Supporting the possibility of a partially redundant function among the three homologues, the attenuated survival of bacteria lacking all three genes was able to be complemented only in part by PagK2 expression *in trans *(Figure 4).

Comparing mRNA levels in Δ*ssrA*/*ssrB *and Δ*phoP*/*phoQ *strains, transcription of *srfN *and *pag *genes (*pagC, pagD*, and *pagK1*/*pagK2*/*pagJ*) was strongly dependent on PhoP, as noted previously [27,38], and to a lesser extent on the SPI-2-encoded two-component regulator SsrA/SsrB, suggesting a positive role of the SPI-2 system in their regulation (Additional file 11, Figure S7). However, the absence of SPI-2 T3SS increased the levels of SrfN and Pag proteins inside macrophages, even though bacterial growth was restrained due to the lack of SPI-2 T3SS (Figure 5). Considering the result that the lack of SPI-2 T3SS did not increase mRNA levels of *srfN *and *pagK1/pagK2*/*pagJ *inside macrophages (Additional file 10, Figure S6), the increase in SrfN and Pag proteins must be a consequence of post-transcriptional regulation. The negative regulation of the translation of *srfN *and *pagK *homologues by SPI-2 T3SS raises the possibility that an activator such as a small non-coding RNA differentially regulates their translation in response to the lack of SPI-2 T3SS secretion following macrophage infection. The unusually long 5'-untranslated region of *srfN *(654-bp upstream of the translation start site; [27]) may form a complex hairpin stem-loop secondary structure that would block the ribosomal binding site and thereby interfere with translation initiation. We observed that deleting N-terminal amino acids in SrfN abolished the negative regulation of SPI-2 T3SS on *srfN *translation and, furthermore, Hfq overexpression increased *srfN *translation, but not its transcription (unpublished data). These observations support the hypothesis that a small RNA, as yet unidentified, might bind to the *srfN *mRNA 5'-region covering the N-terminus and alleviate an inhibitory secondary structure at the translation initiation site, sensing the absence of SPI-2 type III secretion, as is being investigated further.

The T3SS is a typical mechanism for pathogenic bacteria to deliver virulence factors into extracellular environments. However, SrfN and PagK homologues were translocated into the host cytoplasm independently from any T3SSs tested in this study. As a vehicle system to deliver bacterial components to the extracellular milieu, outer membrane vesicles (OMV) have been studied over the past few decades [51-53]. Deatherage *et al*. recently proposed a mechanism for OMV biogenesis wherein envelope protein interconnections modulate OMV release [54]. OMV, observed in a variety of Gram-negative pathogens, are composed of outer membrane proteins, periplasmic proteins, lipopolysaccharide (LPS), and phospholipids and transfer bacterial DNAs and virulence factors to adjacent bacterial or host cells [51,52,55]. PagC was recently found to be secreted extracellularly via OMV as a major component of the outer membrane [56]. The result that PagC was regulated and translocated in a similar manner with SrfN and PagK homologues in our study suggests the possibility of OMV-mediated transfer of SrfN and Pag proteins. In fact, PagK homologues were distributed as punctate compartments apart from intracellular bacteria inside macrophages in microscopic observation, supporting the possibility [57].

## Conclusions

The approach of integrating multiple omics datasets (transcriptomic and proteomic) across multiple regulatory perturbations described in this study generated a robust association network in *Salmonella *highlighting regulatory interactions and provided a precise picture of putative virulence factors. The success of this strategy to discover known and novel virulence factors suggests that the approach may have applicability for other bacterial pathogens, and thereby help to innovate the host-pathogen research paradigm [58].

## Methods

### *Salmonella *strains and growth conditions

All deletion and tagged strains were constructed using *Salmonella enterica *serovar Typhimuirum 14028s as the parent strain. The phase λ Red recombination system was employed to delete or tag genes of interest as described previously [24,59]. Linearized PCR products containing an antibiotic resistance cassette (*kan *or *cat*) and 40-nt sequences, homologous to the target sites, at both termini were introduced into recipient cells to replace genes of interest. FLP recombinase produced from pCP20 eliminated the antibiotic resistance gene via site-specific recombination [59] and resulted in in-frame and non-polar deletions of the target genes. Construction of mutant strains lacking regulators was described in detail previously [24]. Translational fusions of double HA, CyaA' and Bla were constructed in a similar manner using pKD13-2HA [26], pMini-Tn5-cycler [41] and pMini-Tn5-BLAM [8] as PCR template plasmids. A DNA fragment encoding 2HA, CyaA' or Bla as well as Kan was introduced prior to the stop codon sequence of target gene. All primers used in strains construction are listed in Additional file 13, Table S4. Construction of chromosomal *sipA*::*cyaA' *and *sseJ*::*cyaA' *was described previously [41].

pSrfN and pPagK2 expressing SrfN and PagK2 respectively were constructed by cloning DNA fragments containing 900 bp upstream sequences from each start codon and coding sequences of *srfN *or *pagK2 *on pWKS30 via EcoRI and XbaI. Primers used in the PCR amplification of *srfN *and *pagK2 *are shown in Additional file 14, Table S5.

To visualize *Salmonella *using red fluorescent protein (Tomato) in microscopic analysis, bacteria were transformed with pWKS30-Tomato [8].

Bacterial cells were grown in Luria-Bertani (LB) medium or acidic minimal medium (AMM) as described previously [24].

### Transcriptomic analysis

Bacteria were grown under four different conditions: log phase in LB medium, stationary phase in LB medium, MgM dilution (AMM1), and MgM medium shock (AMM2) as described previously [24]. Following RNAprotect (Qiagen) treatment to prevent RNA degradation, cells were lysed and processed to isolate total RNA as described in the instructions of Qiagen RNeasy midi kit (Qiagen) in combination with DNase I (Qiagen) treatment. Isolated total RNA was analysed by RNA 6000 nano assay (Agilent 2100 bioanalyzer, Agilent Technologies). Three biological replicates of RNAs from each strain under each growth condition were pooled with the same amount of RNA from each sample due to the number of microarray necessary for this experiment. A Δ*crp *strain had a growth defect in an acidic minimal medium and thereby was excluded from transcriptional profiling for AMM1 condition. Overall, a total of 59 RNA pools from 15 strains in 4 conditions were subjected to microarray analysis according to the procedures described [60]. Strains tested included fourteen mutant strains lacking virulence regulators and one wild-type strain (*S*. Typhimurium 14028s). Total RNAs from 15 strains were reverse transcribed with random hexamers and Superscript II (Invitrogen) and the subsequent reverse transcripts were labeled by Cy3-linked dCTP (Amersham Biosciences). As the reference panel in all microarray analyses, genomic DNA was isolated from *S*. Typhimurium14028s grown in LB broth overnight using GeneElute bacterial genomic DNA kit (Sigma) and then labeled with Cy5-linked dCTP (Amersham Biosciences). A hybridization mixture containing equivalent amounts of Cy3-labled probes and Cy5-labeled probes was applied on the *Salmonella *array slide and incubated in a hybridization chamber (Corning) at 42°C overnight. The *Salmonella*-specific microarray used in this study represented PCR-amplified sequences from the annotated open reading frames (ORFs) of *S*. Typhimurium LT2 and was further supplemented with annotated ORFs from the serovar Typhi CT18 strain that were > 10% divergent from those of serovar Typhimurium [60]. Each array slide has identical triplicate arrays for statistical analysis. Slides treated with Cy3/Cy5-labled probes were scanned using ScanArray Express (Packard Bioscience, BioChip Technologies) and the fluorescent signal intensities were quantified using QuantArray software (Packard Bioscience, BioChip Technologies) and exported to Excel files. The raw data was processed in WebArray [61] for statistical normalization. The results were displayed in a log_2 _scale (log_2_[signal intensity in a mutant/signal intensity in a wild-type]). Links to raw proteomics and transcriptomics data are available at our project website http://www.Sysbep.org.

### Proteomic analysis: capillary liquid chromatography-mass spectrometry (LC-MS) analysis

#### Soluble Protein Preparation

Cell pellets were resuspended in 100 mM NH_4_HCO_3_, pH 8.4 buffer and lysed by using 0.1 mM zirconia/silica beads in a 2.0-ml Cryovial with vigorous vortexing for a total of 3 min with cooling steps. The supernatant and subsequent washes were transferred from the beads into new Cryovials. The beads were repeatedly washed until the supernatant was clear. After protein concentration was determined for the samples, urea and thiourea were added to final concentrations of 7 and 2 M, respectively. Following addition of DTT (5 mM), the samples were incubated at 60°C for 30 min. The samples were then diluted 10-fold with buffer, and CaCl_2 _was added (1 mM) followed by trypsin in a 1:50 trypsin:protein ratio. The samples were digested for 3 h at 37°C and subsequently cleaned using a C_18 _solid phase extraction (SPE) column (Supelco). Each 1-ml 100- or 50-mg SPE column was conditioned with MeOH and rinsed with 0.1% TFA in water. Samples were introduced to the columns and then washed with 95:5 H_2_O:ACN that contained 0.1% TFA. Excess liquid was removed from the columns under vacuum, and the samples were eluted with 80:20 ACN:H_2_O that contained 0.1% TFA and concentrated in a SpeedVac (Thermo-Savant) to a final volume of ~100 μl. A BCA protein assay was performed to determine peptide concentrations prior to analysis.

#### Insoluble Protein Preparation

Cell pellets were treated and lysed as described above for soluble protein preparations. The lysate was centrifuged at 1,300 × *g *at 4°C for 2 min, and the supernatant was transferred to polycarbonate ultracentrifuge tubes (Beckman) and centrifuged at 4°C at 356,000 × *g *for 10 min. Pellets were resuspended in 50 mM NH4HCO3, pH 7.8, and ultracentrifuged under the same conditions as used in the previous step. A BCA protein assay was performed on the pellets resuspended in water, and the samples were ultracentrifuged once again (as described above) before discarding the supernatant. Pellets were resuspended in ~200 μL of a solubilization solution (7 M urea, 2 M thiourea, 1% CHAPS in 50 mM ammonium bicarbonate, pH 7.8), and DTT was added to a final concentration of 9.7 mM. Samples were incubated at 60°C for 30 min, then diluted, and digested in the same manner as described for the global and soluble protein preparation. Samples were cleaned by using an appropriately sized strong cation exchange (SCX) SPE column (Supelco). Each 1-ml 100-mg column was conditioned with MeOH, rinsed in varying sequences and amounts with 10 mM ammonium formate in 25% ACN, pH 3.0; 500 mM ammonium formate in 25% ACN; and Nanopure water. Samples were acidified to a pH of ≤ 4.0 by adding 20% formic acid followed by centrifugation at 16,000 × *g *for 5 min. Samples were then introduced to the columns and washed with 10 mM ammonium formate in 25% ACN, pH 3.0. Excess liquid was removed from the columns under vacuum. The samples were eluted with 80:15:5 MeOH:H_2_O:NH_4_OH and concentrated to ~100 μl using a SpeedVac. Final peptide concentrations were determined using a BCA protein assay.

#### Capillary Liquid Chromatography (LC)-Mass Spectrometry (MS) Analysis

LC-MS spectra were analyzed using the accurate mass and elution time (AMT) tag approach [30]. Briefly, the theoretical mass and the observed normalized elution time (NET) of each peptide identified by LC-MS/MS was used to construct a reference database of AMT tags, which served as two-dimensional markers for identifying peptides in subsequent high resolution and high mass accuracy LC-MS analyses. A reference database of AMT tags for *S*. Typhimurium has been generated through exhaustive SCX fractionation and LC-MS/MS analysis as described in our previous publications [26] and the LC-MS/MS data from this experiment, obviating the need for further time consuming SCX LC-MS/MS analyses. This approach to proteomics research was enabled by a number of published and unpublished in-group developed tools, which are available for download at http://omics.pnl.gov[62-66]. Prior to the samples being analyzed they were subjected to a blocking and randomization treatment to minimize the effects of systematic biases and ensure the even distribution of known and unknown confounding factors across the entire experimental dataset. Peptides from each of the soluble and insoluble protein preparations were separated by an automated in-house designed reverse-phase capillary HPLC system as described elsewhere [67]. Eluate from the HPLC was directly electrosprayed into an LTQ-Orbitrap mass spectrometer (LTQ-Orbitrap, Thermo Fisher Scientific) using electrospray ionization (ESI) with emitters described previously [68] and the ESI interface modified with an electrodynamic ion funnel [69]. Three biological replicates for each sample were pooled and analyzed on the ESI interface modified LTQ-Orbitrap mass spectrometer. Relevant information such as the elution time from the capillary LC column, the abundance of the signal (integrated area under the peptide peak), and the monoisotopic mass (determined from charge state and the high accuracy *m*/*z *measurement) of each feature observed in the LTQ-Orbitrap was used to match the peptide identifications contained within the AMT tag database. A matching feature was required to be within 1.6 ppm of the peptide mass and the normalized elution time (NET) within 2%. To assess false discovery rates each feature observed in the LTQ-Orbitrap was used to match the peptide identifications contained within a decoy AMT tag database. An FDR of < 1% was calculated. The abundances of these identified peptides are quantified using the area under the peptide peak and were used to infer the protein composition and relative protein abundances as described [70] and implemented in the software program DAnTE [71]. Links to raw proteomics and transcriptomics data are available at our project website http://www.Sysbep.org.

### Network analysis of omics data

The context likelihood of relatedness (CLR) algorithm [23] was utilized to systematically infer association relationships between proteins or genes. For the proteomics network we used the LC-MS-derived abundance profiles (as described above) from all the deletions under each of the four conditions with missing values filled with values corresponding to one-half the minimal observed abundance value as input to CLR. CLR calculates the mutual information between all pairs of proteins and then calculates a normalized relationship score for all resulting edges. We used 5 bins for calculation of mutual information in CLR and a spline degree of 3 for curve fitting of the binned data. For the transcriptomic network we used the transcriptional data described above as input into CLR.

To expand the integrated network to include genes not observed by proteomics, we used an approach similar to that used for topological analysis of the integrated networks (Additional file 2, Methods S1 and Additional file 5, Table S3). Each network was first filtered individually to retain only highly confident edges. The proteomics network was filtered to include only edges with Z scores above 6.0 and the transcriptomics network was filtered to include edges with Z scores above 8.0. A more stringent threshold was used with the transcriptomics to limit the influence of the much larger dataset and retain a focus on the proteomics data. Other thresholds were also applied and these resulted in very similar clustering. These networks also retained the enrichment of bottlenecks in virulence essential genes, observed in the integrated network above (data not shown). Clustering was accomplished by considering the final expanded integrated network as a similarity matrix, with a value of 0 for those gene/protein pairs that did not have an edge in the final network. The similarity matrix was used as input to the hclust function in R using the mcquitty agglomeration method [72].

### Macrophage infection and cAMP assay

*Salmonella *cells were grown in Luria-Bertani (LB) broth and then opsonized with 1% mouse serum (Innovative Research) for 20 min prior to infection. RAW264.7 cells, murine macrophage-like cells (ATCC TIP-71), were infected with *Salmonella *at an input multiplicity of infection (MOI) of 100 by centrifuging the bacteria onto the macrophage monolayers at 1,000 × g for 5 min. The conditioned Dulbecco's Modified Eagle's Medium (DMEM) was replaced with DMEM containing gentamycin (Gibco) at 100 μg/ml to remove extracellular bacteria following 30 min incubation at 37°C with 5% CO_2_. After gentamycin (100 μg/ml) treatment for 1 hour, cells were washed with PBS and overlaid with DMEM containing 20 μg/ml gentamycin for the remainder of the experiments. To examine translocation of *Salmonella *proteins into the cytosol, macrophage cells were washed with PBS and then lysed with 0.1 M HCl as directed in the manufacturer's instructions for the cyclic AMP EIA kit (Assay Designs). Macrophage debris containing bacteria was removed by centrifugation and the cytosolic fraction was used to determine cAMP levels in the cytoplasm.

### Immunoblot analysis

To analyze proteins from *Salmonella *grown in laboratory conditions, cell lysate from 5 × 10^7 ^colony-forming units (CFU) was loaded on SDS-PAGE gel. For Western blotting on intracellular *Salmonella *cells, infected macrophage cells were disrupted with a lysis buffer (50 mM HEPES, 1 mM EDTA, 1 mM EGTA, 1% Triton X-100, 100 mM PMSF, protease inhibitor cocktail (Roche), 50 mM NaF, DNase I (Qiagen; DNase Set), and 2 mM sodium orthovanadate) on ice for 10 min and centrifuged at 10,000 × g for 10 min to pellet intracellular bacteria. The pellet was directly resuspended in Laemmli sample buffer, boiled for 5 min, and then loaded on SDS-PAGE gel. Primary antibodies used are anti-HA.11 antibody (Covance), ant-CyaA (3D1) antibody (Santa Cruz Biotechnology), and anti-DnaK antibody (Assay Designs). As a secondary antibody, anti-mouse IgG conjugated with peroxidase (Sigma) was used in all immunoblot experiments.

### CCF4-AM cleavage detection

To detect translocation of Bla fusions, macrophages were seeded in Lab-Tek II chamber coverglass slides (Nunc) and infected with *Salmonella *Bla fusion strains as described above. CCF4-AM (Invitrogen) solution was loaded on macrophages monolayers for 2 hours per manufacturer's instructions and the cleavage was analyzed using an Applied Precision (Issaquah) DeltaVision image restoration system with emission filter sets for green (528 nm) and blue (457 nm) fluorescence by CCF4-AM and Tomato fluorescent protein (617 nm). CCF4-AM cleavage assays were performed three times and the images shown are representatives of three independent assays.

### Mouse infection experiments

In order to estimate competitive index for each test strain, *Salmonella *mutant/wild-type strains and the reference strain MA6054 [73] were cultivated in LB medium overnight. *Salmonella *Typhimurium MA6054 is a genetically manipulated strain expressing β-galactosidase in the presence of L-arabinose. A mixture of cells containing equivalent numbers from each test strain and the reference strain was inoculated into five female 129SvJ mice of 5 weeks old at 10,000 CFU/mouse. Mice were dissected to isolate spleens at 7 days post-infection. The spleen homogenate and the inoculum were plated on LB agar medium containing 40 μg/ml X-gal (5-bromo-4-chloro-3-indolyl-β-D-galactopyranoside) and 1 mM arabinose. The competitive index (CI) was calculated as [% of test strain recovered/% of reference strain recovered]/[% of test strain inoculated/% of reference strain inoculated].

## Authors' contributions

HY, CA, JEM, FH, and JNA conceived and designed the experiments. HY, CA, and MG performed the laboratory experiments. HY, CA, JEM, FH, and JNA analyzed and interpreted the data and results. JEM and RDS contributed reagents, materials, and/or analysis tools. HY, CA, JEM, FH, and JNA wrote the paper. JNA, FH, and RDS provided grant support. All authors have read and approved the final manuscript.

## Supplementary Material

Additional file 1 **Table S1. Proteins quantified by mass spectrometry**. Click here for file

Additional file 2 **Methods S1**. Click here for file

Additional file 3 **Figure S1**. Integration of networks inferred from transcriptomics and proteomics improves enrichment of genes essential for virulence in *Salmonella*. Click here for file

Additional file 4 **Table S2**. Topological enrichment in protein association and integrated networks. Click here for file

Additional file 5 **Table S3**. Network topology of integrated network. Click here for file

Additional file 6 **Figure S2**. Coordinate regulation of proteomics-identified novel effectors Click here for file

Additional file 7 **Figure S3**. Expression of candidate proteins under *in vitro *and *ex vivo *conditions. Click here for file

Additional file 8 **Figure S4**. Sequence alignment of *pagJ, pagK*, and STM2585A. Click here for file

Additional file 9 **Figure S5**. Translocation of SseJ into the macrophage cytosol. Click here for file

Additional file 10 **Figure S6**. Translocation of SipA and SseJ in macrophages infected with Δ*invA *and Δ*ssaK *strains. Click here for file

Additional file 11 **Figure S7**. Effects of SPI-2 TTSS on the transcription of *srfN*, and *pagJ*/*pagK1*/*pagK2 *inside macrophages. Click here for file

Additional file 12 **Figure S8**. Effects of PhoP/PhoQ and SsrA/SsrB on the transcription of *srfN *and *pag *genes. Click here for file

Additional file 13 **Table S4**. Primers used in strains construction. Click here for file

Additional file 14 **Table S5**. Primers used in qRT-PCR. Click here for file
