# Supplementary material for: Systems analysis of multiple regulator perturbations allows discovery of virulence factors in Salmonella
Source: BMC Syst Biol. 2011 Jun 28;5:100. doi: 10.1186/1752-0509-5-100 (PMC3213010; doi:10.1186/1752-0509-5-100)
Supplement: Additional file 2 — Methods S1. [file 1752-0509-5-100-S2.PDF]

Additional file 2

Supplementary Methods

Topological analysis of integrated transcriptomic and proteomic association networks

In this study we describe a novel application of the existing context likelihood of relatedness (CLR) method [1] to global proteomics data to define protein association networks. It is important to note that we are not applying CLR for its originally intended purpose of inferring regulatory networks (relationships between transcription factors), but rather to identify pairs of proteins that have abundance profiles that are significantly more similar than background, where we define the background as all other pairwise comparisons between proteins in the network. The result is a co-abundance network in which edges represent several different possibilities: one protein may regulate the abundance of the other, both proteins may be co-regulated because they are in the same complex or functional pathway, or the two proteins may be coincidentally co-abundant over the conditions examined. Coincidental co-abundance (a false positive prediction) becomes less likely as the stringency of the edge threshold is increased and the number of conditions considered for the mutual information is increased. In our application we believe that the number of coincidental edges is likely to be low, because the thresholds used are quite stringent, there are a large number of conditions (63 considered), and the definition of a ‘true positive’ association is quite broad. It is difficult or impossible to establish a false positive discovery rate for the network precisely because true positive associations are broadly defined and there are no datasets that would encompass all true positive associations. However, this consideration does not impact the results presented

in the main text or this supplement because in the topological analysis (described below) we are using the network to identify virulence-related genes and can show that this is successful, and in the main text we validate associations experimentally.

The proteomic data-inferred network was analyzed to identify topology for each protein as described previously [2], over a range of CLR Z score thresholds. We assessed the enrichment of the top 20% of proteins ranked by degree (hubs) and betweenness (bottlenecks) in a list of genes identified as being essential for virulence in negative genetic screens [3-6] using a Fisher's exact test. However, the topological analysis of proteomics-derived network showed that there was little or no enrichment in hubs or bottlenecks for virulence essential gene products (Additional file 4, Supplementary Table S2).

In a previous study we showed that integrating networks inferred from proteomics with other kinds of networks increased the importance of highly central proteins, strengthening the veracity of computational inference [7]. We therefore integrated the proteomics-inferred network with the transcriptomics-inferred network by considering the intersection of nodes (proteins or genes) in each network and averaging the weights for each pair of nodes. This resulted in a combined network with the same number of nodes as the original proteomics network, which we filtered to include high-confidence edges for association (Z score > 3.0). Topological enrichment analysis was compared between the combined network and the two 'parent' networks, employing bottleneck genes/proteins filtered at three different thresholds (Additional file 3, Figure S1A). Essential virulence genes/proteins were moderately enriched when the top 20% of nodes was evaluated, showing comparable enrichment among three inferred

networks. However, bottlenecks with more stringent thresholds (2.5% or 10%) were more significantly enriched in the combined network than in either parent network, indicating that topology of the combined network provides improved ordering of important genes/proteins. When the topology analysis was applied to bottleneck proteins with homology to *Escherichia coli* (protein identity > 95%) and mouse (protein identity > 20%) proteins, the combined network also showed high enrichment (Additional file 4, Table S2). Virulence genes/proteins ranked by topological degree (hubs) showed a tendency to be enriched in the combined network as well, although at the highest degree stringency (2.5%) the proteomics network was highly enriched in virulence essential proteins (Additional file 3: Supplementary Figure S1B). Though the p-values calculated for topological enrichment were not extremely low due to the limited number of bottlenecks/hubs being considered, these are promising results that build upon our previous results reported in the full transcriptomic network.

Topological analysis of the resulting networks was performed using the igraph library in R, and statistical enrichment of bottlenecks and hubs was performed in R using the Fisher's exact test. Genes identified in at least two of the five negative genetic screens [3-6] for virulence were considered to be essential in virulence, a total of 82 genes in the network (see Additional file 5: Supplementary Table S3, VirSum column). Bottlenecks and hubs were initially defined as the top 20% of genes or proteins as ranked by betweenness or degree, respectively. However, making the set of bottlenecks more conservative by considering the top 10% or 2.5% improved enrichment results as seen in Additional file 7 (Additional file 3, Supplementary Figure S1A).

Inferred transcriptomics and proteomics networks were integrated by calculating the mean of the CLR Z scores from each network for all proteins observed in the proteomics data (1349 proteins; **Additional file 1, Table S1**). The resulting matrix was then filtered to remove relationships with mean Z scores less than 3.0 for the final analysis, though other thresholds produced similar results (data not shown).

Quantitative real-time PCR

For RNA preparation under *in vitro* conditions, *Salmonella* cells were grown in AMM1 condition as described and harvested for RNA extraction. Total RNA was isolated using RNAprotect, RNeasy kit, and DNase set (all from Qiagen) according to the manufacturer's instructions. To analyze gene expression inside macrophages, RAW264.7 cells were infected with *Salmonella* as described and RNA degradation was quenched by RNAlater (Ambion) at 18 h post-infection. Macrophage cells were lysed in a lysis buffer (50 mM HEPES, 1 mM EDTA, 1 mM EGTA, 1% Triton X-100, 100 mM PMSF, protease inhibitor cocktail (Roche), 50 mM NaF, and 2 mM sodium orthovanadate) on ice for 20 min and bacterial cells were isolated by centrifugation at 10,000×g for 10 min. Pelleted bacterial cells were processed following the instructions of RNeasy kit and DNase set to isolate total RNA. cDNA was synthesized using iScript cDNA synthesis kit (Bio Rad) and subjected to quantitative real-time PCR (StepOnePlus real-time PCR systems, Applied Biosystems) with Power SYBR green PCR master mix (Applied Biosystems) and primer sets. Primers were designed using Primer Express software v3.0 (Applied Biosystems). mRNA levels were normalized with *gyrB* mRNA and the expression fold was estimated. *gyrB* is assumed to be a steadily transcribed housekeeping gene and has been used as a reference gene [2, 8]. The expression fold is the average

from at least three independent RNA samples. Primers used in this assay are listed in Additional file 14, Table S5.

References

1. Faith JJ, Hayete B, Thaden JT, Mogno I, Wierzbowski J, Cottarel G, Kasif S, Collins JJ, Gardner TS: **Large-scale mapping and validation of Escherichia coli transcriptional regulation from a compendium of expression profiles.** *PLoS Biol* 2007, **5**:e8.

2. Yoon H, McDermott JE, Porwollik S, McClelland M, Heffron F: **Coordinated regulation of virulence during systemic infection of Salmonella enterica serovar Typhimurium.** *PLoS Pathog* 2009, **5**:e1000306.

3. Chan K, Kim CC, Falkow S: **Microarray-based detection of Salmonella enterica serovar Typhimurium transposon mutants that cannot survive in macrophages and mice.** *Infect Immun* 2005, **73**:5438-5449.

4. Lawley TD, Chan K, Thompson LJ, Kim CC, Govoni GR, Monack DM: **Genome-wide screen for Salmonella genes required for long-term systemic infection of the mouse.** *PLoS Pathog* 2006, **2**:e11.

5. Morgan E, Campbell JD, Rowe SC, Bispham J, Stevens MP, Bowen AJ, Barrow PA, Maskell DJ, Wallis TS: **Identification of host-specific colonization factors of Salmonella enterica serovar Typhimurium.** *Mol Microbiol* 2004, **54**:994-1010.

6. Shah DH, Lee MJ, Park JH, Lee JH, Eo SK, Kwon JT, Chae JS: **Identification of Salmonella gallinarum virulence genes in a chicken infection model using PCR-based signature-tagged mutagenesis.** *Microbiology* 2005, **151**:3957-3968.

7. Diamond DL, Syder AJ, Jacobs JM, Sorensen CM, Walters KA, Proll SC, McDermott JE, Gritsenko MA, Zhang Q, Zhao R, Metz TO, Camp DGn, Waters KM, Smith RD, Rice CM, Katze MG: **Temporal proteome and lipidome profiles reveal hepatitis C virus-associated reprogramming of hepatocellular metabolism and bioenergetics.** *PLoS Pathog* 2010, **6**:e1000719.

8. Navarre WW, Porwollik S, Wang Y, McClelland M, Rosen H, Libby SJ, Fang FC: **Selective silencing of foreign DNA with low GC content by the H-NS protein in Salmonella.** *Science* 2006, **313**:236-238.
