# Supplementary material for: Systems analysis of multiple regulator perturbations allows discovery of virulence factors in Salmonella
Source: BMC Syst Biol. 2011 Jun 28;5:100. doi: 10.1186/1752-0509-5-100 (PMC3213010; doi:10.1186/1752-0509-5-100)
Supplement: Additional file 3 — Figure S1. Integration of networks inferred from transcriptomics and proteomics improves enrichment of genes essential for virulence in Salmonella. [file 1752-0509-5-100-S3.PDF]

Additional file 3

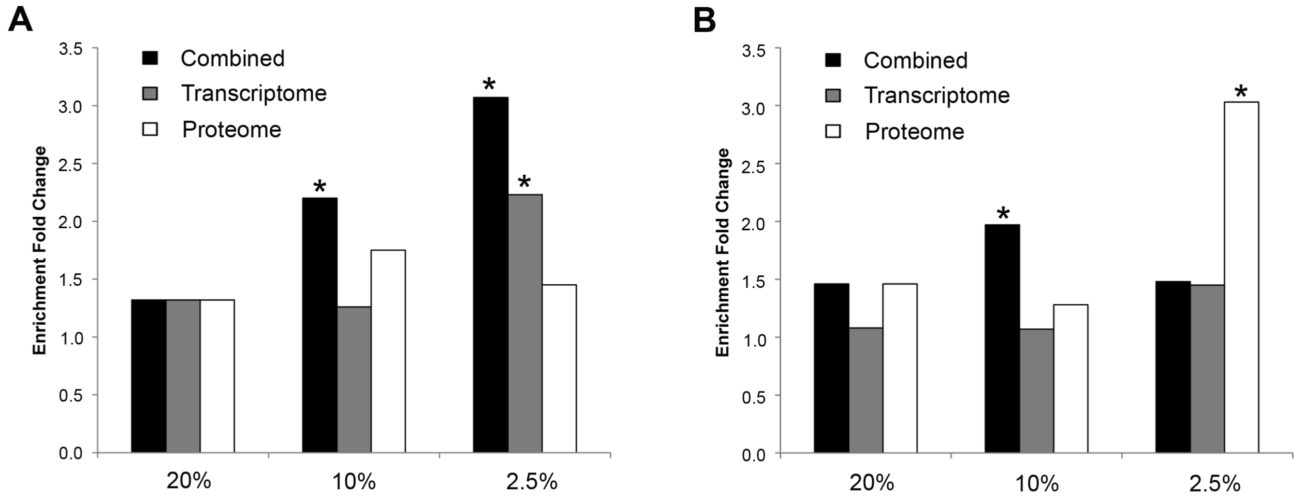

Supplementary Figure S1. Integration of networks inferred from transcriptomics and proteomics improves enrichment of genes essential for virulence in *Salmonella*.

Association networks were inferred from mRNA expression profiles (transcriptome) or protein abundance levels (proteome) using CLR. The resulting networks were integrated (combined) by calculating the mean of edge Z scores. **A.** Statistical enrichment in genes essential for virulence was calculated in the highest portion (compare bottleneck thresholds of 20, 10, and 2.5%) of nodes (proteins or genes) ranked by topological betweenness, and a fold increase over background was calculated (fold enrichment). **B.** Topological enrichment of nodes ranked by topological degree.
