# Supplementary material for: Systems analysis of multiple regulator perturbations allows discovery of virulence factors in Salmonella
Source: BMC Syst Biol. 2011 Jun 28;5:100. doi: 10.1186/1752-0509-5-100 (PMC3213010; doi:10.1186/1752-0509-5-100)
Supplement: Additional file 6 — Figure S2. Coordinate regulation of proteomics-identified novel effectors [file 1752-0509-5-100-S6.PDF]

Additional file 6

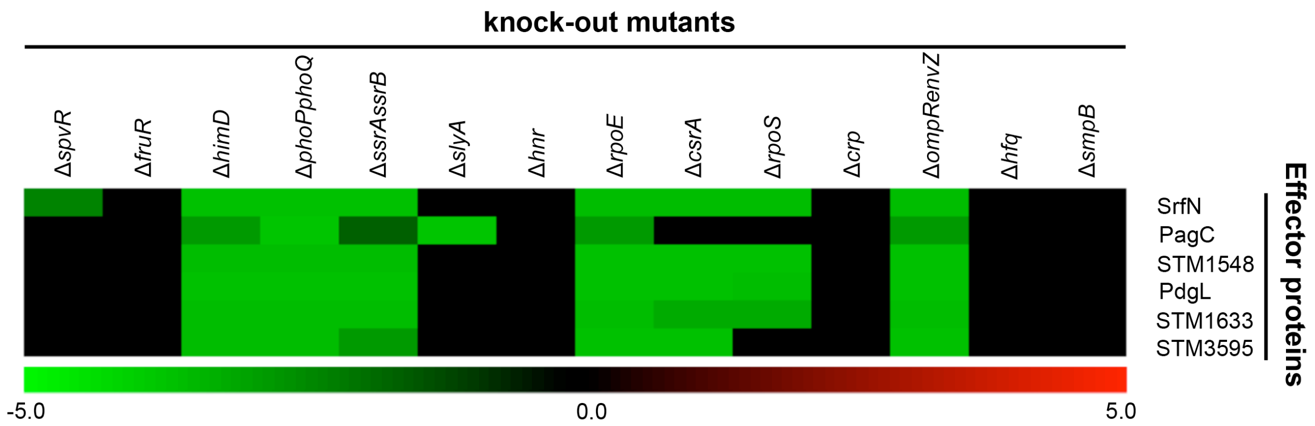

Supplementary Figure S2. Coordinate regulation of proteomics-identified novel effectors.

Analysis of the extent to which deleting a particular regulator affected the abundance of proteomics-detected novel effectors under infectious-like state (acidic minimal media condition; AMM1). Data visualized are log_2-transformed fold difference between a knock-out mutant and wild-type. Scale ranges from fold decrease relative to wild-type (green) to fold increase relative to wild-type (red).
