# Supplementary material for: Systems analysis of multiple regulator perturbations allows discovery of virulence factors in Salmonella
Source: BMC Syst Biol. 2011 Jun 28;5:100. doi: 10.1186/1752-0509-5-100 (PMC3213010; doi:10.1186/1752-0509-5-100)
Supplement: Additional file 7 — Figure S3. Expression of candidate proteins under in vitro and ex vivo conditions. [file 1752-0509-5-100-S7.PDF]

Additional file 7

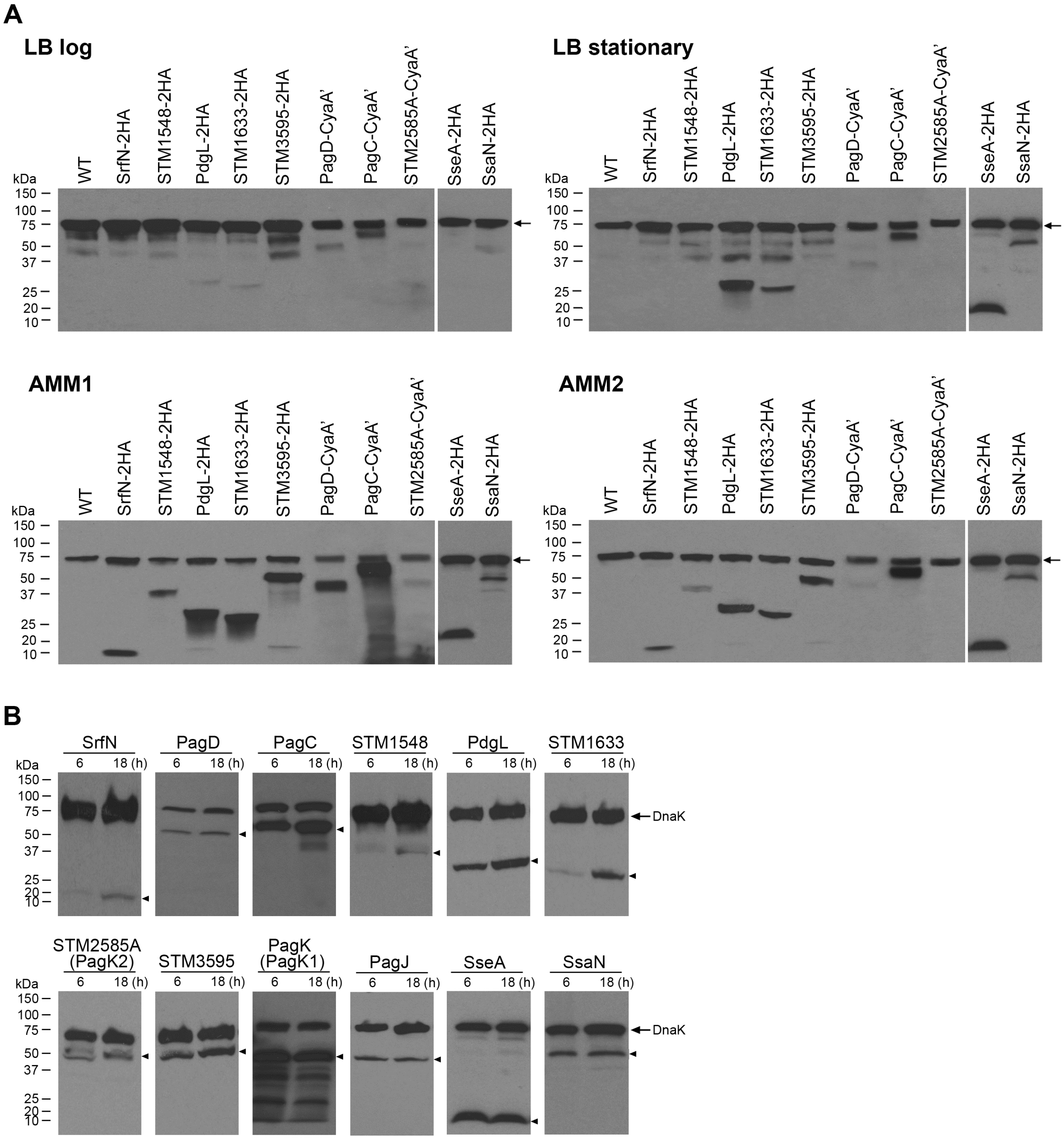

Supplementary Figure S3. Expression of candidate proteins under *in vitro* and *ex vivo* conditions.

A. To validate the results of transcriptomic and proteomic analyses, eight candidate proteins were tagged with double HA or CyaA' and their expression was examined in 4 *in vitro* conditions; log phase in LB, stationary phase in LB, AMM1, and AMM2. SPI-2 proteins (SseA and SsaN) were tagged with double HA and their expression was compared in 4 conditions in parallel. Bands at 70 kDa indicate DnaK, which was used to normalize protein amounts between lanes.

B. Candidate proteins and two SPI-2 proteins (SseA and SsaN) were labeled with 2HA or CyaA' and RAW264.7 cells were infected with HA or CyaA'-tagged strains. Expression of tagged proteins inside macrophages was examined using Western blotting at 6 and 18 h post-infection. HA or CyaA'-tagged proteins are indicated by arrowheads. DnaK was used to normalize the protein levels between lanes and indicated by an arrow.
