# Supplementary material for: Systems analysis of multiple regulator perturbations allows discovery of virulence factors in Salmonella
Source: BMC Syst Biol. 2011 Jun 28;5:100. doi: 10.1186/1752-0509-5-100 (PMC3213010; doi:10.1186/1752-0509-5-100)
Supplement: Additional file 8 — Figure S4. Sequence alignment of pagJ, pagK, and STM2585A. [file 1752-0509-5-100-S8.PDF]

Additional file 8

A

```
pagJ      1   ---------------------------atgaaacacgttaagagcgtatttttagcaatggctttaatat  43
pagK      1   ---------------------------atgaaacacgtcaagagcgtatttttagcaatggttttaatat  43
STM2585A  1   gtgccaaaatttaacaggcaacgtattattaaacacgttaagagcgtatttttagcaatgatttaatat  70
                                         ** ******** *********************  ********

pagJ      44  taccatcctcactatattctgctcttacaatagcggcagactccaagatcagcaaaaagcagaaacaat  113
pagK      44  taccatcttcactatatcctgctcttacaatagcggcagactctcaagatcataaaaaagaagaaacaat  113
STM2585A  71  taccatcttcactatattctgctcttacaatagcggcagactctcaagatcataaaaaagaagaaacaat  140
              ******* ********* ************************* ********  ****** *********

pagJ      114 taagcccataccacctaagatgtgtagtctttggcctgcggacgtgcccttccccgaagattggtttaaa  183
pagK      114 taagccaatgcctcaaaagtggtgtaatctttggcctgctggcatacccttccctgaagattggtttaaa  183
STM2585A  141 taagccaatgcctcaaaagtggtgtaatctttggcctgctggcatacccttccctgaagattggtttaaa  210
              ****** ** ** *  ***  ***** ************ * * * ******** ***************

pagJ      184 atgtgtagaggttattga  201
pagK      184 atgtgtagaggttattga  201
STM2585A  211 atgtgtagaggttattga  228
              ******************
```

B

```
PagJ      1 ---------MKHVKSVFLAMALILPSSLYSALTIAADSQDQQKAETIKPIPPKMCSLWPADVPFPEDWFKMCRGY 66
PagK      1 ---------MKHVKSVFLAMVLILPSSLYPALTIAADSQDHKKEETIKPMPQKWCNLWPAGIPFPEDWFKMCRGY 66
STM2585A  1 MPKFNRQRIIKHVKSVFLAMILILPSSLYSALTIAADSQDHKKEETIKPMPQKWCNLWPAGIPFPEDWFKMCRGY 75
                     :********** ********.**********::* *****:* * *.****.:*************
```

Supplementary Figure S4. Sequence alignment of *pagJ*, *pagK*, and STM2585A.

Sequences of three *pagK* homologues were compared and aligned using Mafft v. 6 (http://align.bmr.kyushu-u.ac.jp/mafft/software/). Sequences from *S*. Typhimurium 14028s genome (GeneBank: CP001363) were used: *pagJ* (STM14_1480); *pagK* (STM14_2265); STM2585A (STM14_3167). Identical sequence among three homologues is indicated with asterisk. **A**, nucleic acids alignment; **B**, amino acids alignment.
