# Supplementary material for: Systems analysis of multiple regulator perturbations allows discovery of virulence factors in Salmonella
Source: BMC Syst Biol. 2011 Jun 28;5:100. doi: 10.1186/1752-0509-5-100 (PMC3213010; doi:10.1186/1752-0509-5-100)
Supplement: Additional file 9 — Figure S5. Translocation of SseJ into the macrophage cytosol. [file 1752-0509-5-100-S9.PDF]

Additional file 9

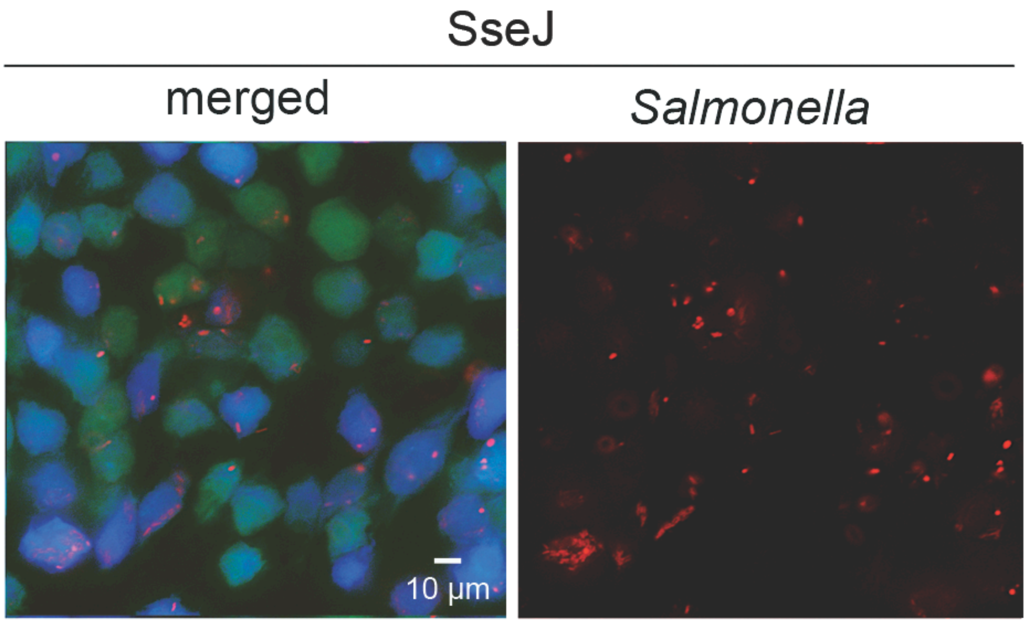

Supplementary Figure S5. Translocation of SseJ into the macrophage cytosol.

SseJ, an effector translocated by SPI-2 TTSS, was labeled with β-lactamase and RAW264.7 cells were infected with *Salmonella* expressing SseJ-Bla for 18 hours. Cells were loaded with CCF4-AM for 2 hours. CCF4-AM cleaved by translocated Bla-tagged proteins changed emission wavelength from 528 nm (green) to 457 nm (blue). Bacteria were transformed with pWKS30-Tomato and shown in red.
