# Supplementary material for: Systems analysis of multiple regulator perturbations allows discovery of virulence factors in Salmonella
Source: BMC Syst Biol. 2011 Jun 28;5:100. doi: 10.1186/1752-0509-5-100 (PMC3213010; doi:10.1186/1752-0509-5-100)
Supplement: Additional file 10 — Figure S6. Translocation of SipA and SseJ in macrophages infected with ΔinvA and ΔssaK strains. [file 1752-0509-5-100-S10.PDF]

Additional file 10

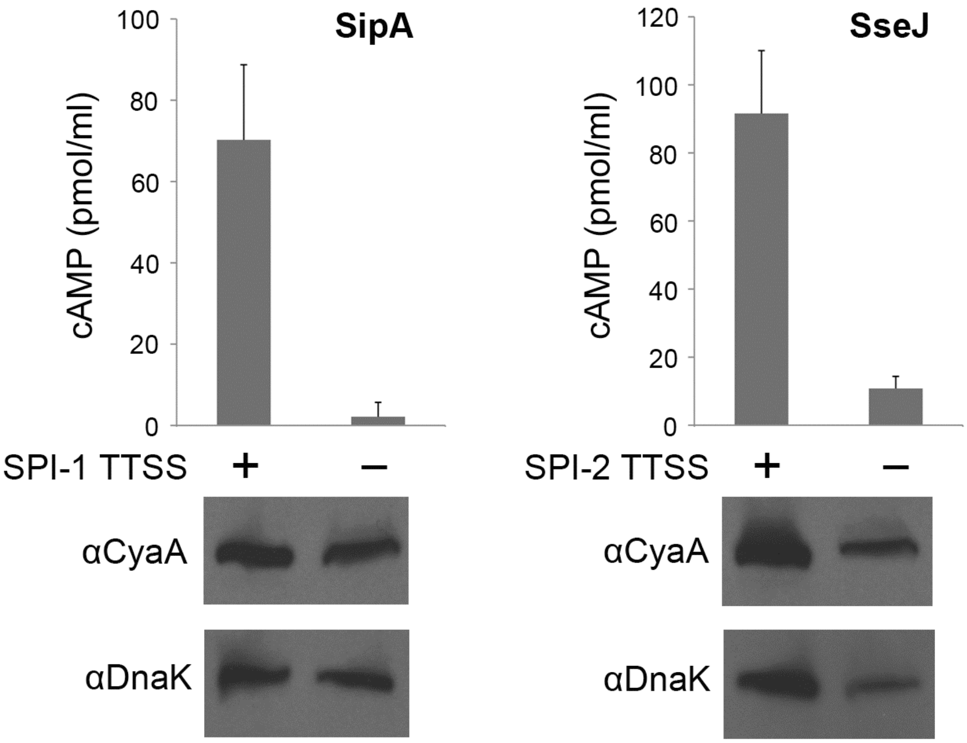

Supplementary Figure S6. Translocation of SipA and SseJ in macrophages infected with Δ*invA* and Δ*ssaK* strains.

In order to verify that SPI-1 T3SS and SPI-2 T3TT were blocked by deletion of *invA* and *ssaK* respectively, *sipA* (SPI-1 T3SS-secreted effector) and *sseJ* (SPI-2 T3SS-secreted effector) were tagged with *cyaA'* in Δ*invA* and Δ*ssaK* respectively and their translocation (top, cAMP assay) and expression (bottom, Western blotting) was examined in parallel. *Salmonella* strains harboring *sipA*::*cyaA'* allele were cultivated in LB for 3 hours to induce SPI-1 expression, and then added to macrophages at an input MOI of 50. Translocation and expression of SipA-CyaA' was measured at 1 h post-infection. Infection with *sseJ*::*cyaA'* strains was performed as described in Methods, and translocation and expression of SseJ-CyaA' fusions was examined at 18 h post-infection. DnaK was used to normalize protein amounts between lanes in Western blotting analysis.
