# Supplementary material for: Systems analysis of multiple regulator perturbations allows discovery of virulence factors in Salmonella
Source: BMC Syst Biol. 2011 Jun 28;5:100. doi: 10.1186/1752-0509-5-100 (PMC3213010; doi:10.1186/1752-0509-5-100)
Supplement: Additional file 11 — Figure S7. Effects of SPI-2 TTSS on the transcription of srfN, and pagJ/pagK1/pagK2 inside macrophages. [file 1752-0509-5-100-S11.PDF]

Additional file 11

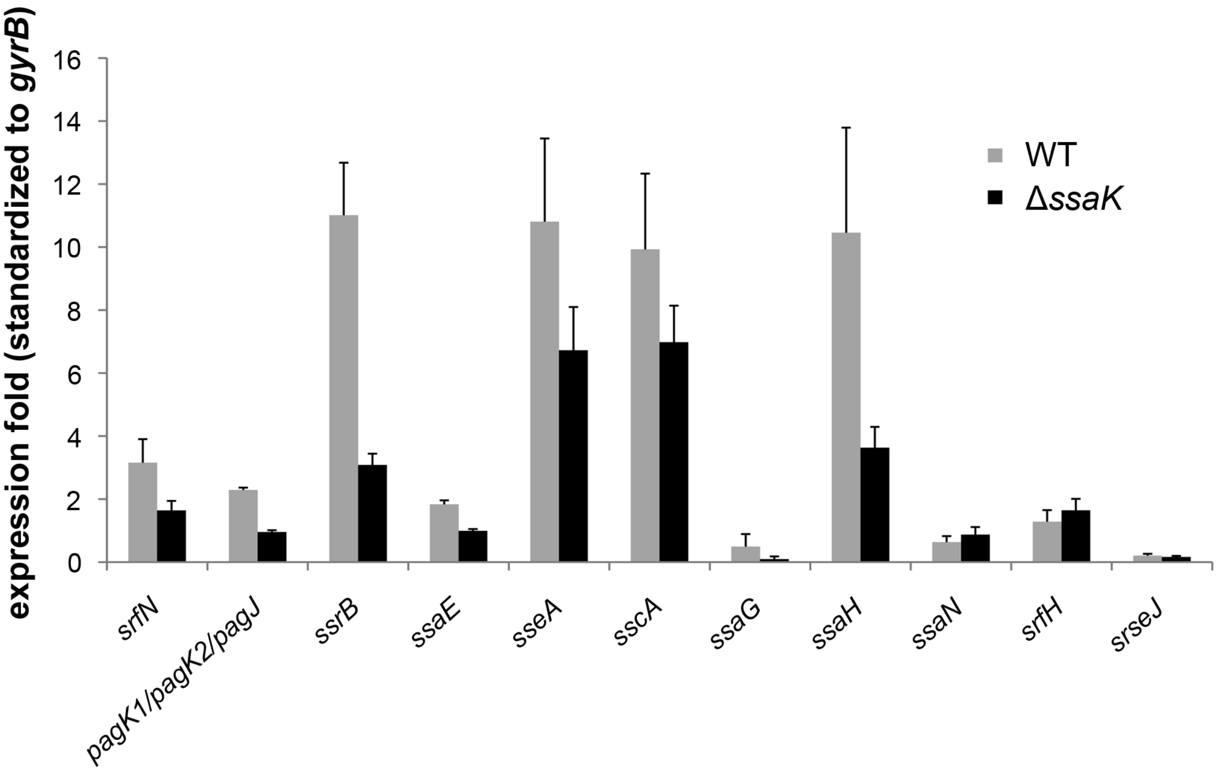

Supplementary Figure S7. Effects of SPI-2 TTSS on the transcription of *srfN*, and *pagJ*/*pagK1*/*pagK2* inside macrophages.

RAW264.7 cells were infected with a wild-type and Δ*ssaK* strains for 18 hours and bacterial total RNAs were isolated to measure transcription levels of a variety of genes including *srfN* and *pagJ*/*pagK1*/*pagK2*. The expression level was normalized with *gyrB* and expression folds relative to *gyrB* were averaged from three independent RNA isolates. *pagJ*, *pagK1*, and *pagK2* were indistinguishable due to the high sequence homology.
