# Supplementary material for: Systems analysis of multiple regulator perturbations allows discovery of virulence factors in Salmonella
Source: BMC Syst Biol. 2011 Jun 28;5:100. doi: 10.1186/1752-0509-5-100 (PMC3213010; doi:10.1186/1752-0509-5-100)
Supplement: Additional file 12 — Figure S8. Effects of PhoP/PhoQ and SsrA/SsrB on the transcription of srfN and pag genes. [file 1752-0509-5-100-S12.PDF]

Additional file 12

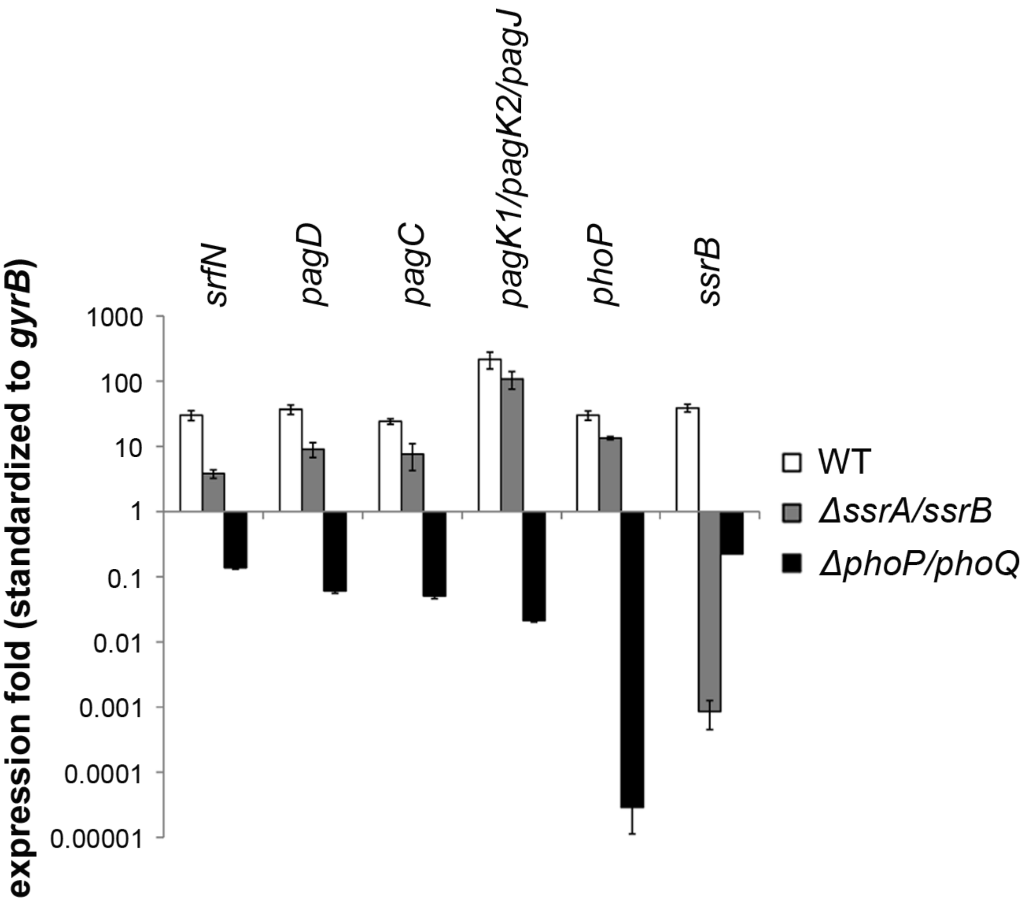

Supplementary Figure S8. Effects of PhoP/PhoQ and SsrA/SsrB on the transcription of *srfN* and *pag* genes.

Total RNAs were prepared from wild-type, Δ*ssrA*/*ssrB*, and Δ*phoP*/*phoQ* strains cultivated in AMM1 condition and subjected to qRT-PCR as described in Supplementary Methods. mRNA levels of *srfN*, *pagD*, *pagC*, *pagK1*/*pagK2*/*pagJ*, *phoP*, and *ssrB* were measured in triplicate using three independent RNA isolates and normalized based on *gyrB* mRNA. The expression fold of a gene to *gyrB* was averaged and plotted. Three *pagK*-homologous genes were indistinguishable each other due to the high sequence homology among them.
