# Supplementary material for: Systems analysis of multiple regulator perturbations allows discovery of virulence factors in Salmonella
Source: BMC Syst Biol. 2011 Jun 28;5:100. doi: 10.1186/1752-0509-5-100 (PMC3213010; doi:10.1186/1752-0509-5-100)
Supplement: Additional file 13 — Table S4. Primers used in strains construction. [file 1752-0509-5-100-S13.PDF]

Additional file 13

Supplementary Table S4. Primers used in strains construction

Name	Sequences (5’ to 3’)	Use
STM0082-HAF1	TTTTGAAGCCGTTGCCGAAGTTTATAACGATGCGACGAAAATTCCGGGGATCCGTCGACC	STM0082-2HA
STM0082-HAR1	TTCATGCCAGTGCCCTCGTTATGGCGCAATCAGAGCTTTAGTGTAGGCTGGAGCTGCTCC	STM0082-2HA
STM1548-HAF1	TGGTGATTGCATGTTAATCATACAAGATGAAGGGCAGGGAATTCCGGGGATCCGTCGACC	STM1548-2HA
STM1548-HAR1	AAAACCGGTAGCATTATATGTACCGGTTTTATTTTATTCAGTGTAGGCTGGAGCTGCTCC	STM1548-2HA
STM1599-HAF1	CCCGAACACTTGGTTTGACTTTCCCGTTAAGCAGAGACCCATTCCGGGGATCCGTCGACC	STM1599-2HA
STM1599-HAR1	CAGGCGTAATGATTAGATTTCGCAACAAAACGCCGTTTCAGTGTAGGCTGGAGCTGCTCC	STM1599-2HA
STM1633-HAF1	GCCACAAAAAATATCTGGCATTGTCGATAGCTGGATGAAAATTCCGGGGATCCGTCGACC	STM1633-2HA
STM1633-HAR1	AAAAGATAGCCAGCATATTACCTGATGACATAGCTTACTAGTGTAGGCTGGAGCTGCTCC	STM1633-2HA
STM3595-HAF1	GGATCTGTCCGCTGCTTACGAGATGGCGAGAAAAACGCGCATTCCGGGGATCCGTCGACC	STM3595-2HA
STM3595-HAR1	TCATCCCGGCTTACGTTTTATAAGCCGGGTGACAACGTCAGTGTAGGCTGGAGCTGCTCC	STM3595-2HA
STM0082-CyaAF1	TTTGAAGCCGTTGCCGAAGTTTATAACGATGCGACGAAACTGTCTCTTATACACATCTCA	STM0082-CyaA’
STM0082-CyaAR1	CATGCCAGTGCCCTCGTTATGGCGCAATCAGAGCTTTA CTGTCTCTTATACACATCTGGT	STM0082-CyaA’
STM1548-CyaAF1	GGTGATTGCATGTTAATCATACAAGATGAAGGGCAGGGACTGTCTCTTATACACATCTCA	STM1548-CyaA’
STM1548-CyaAR1	AACCGGTAGCATTATATGTACCGGTTTTATTTTATTCACTGTCTCTTATACACATCTGGT	STM1548-CyaA’
STM1599-CyaAF1	CCGAACACTTGGTTTGACTTTCCCGTTAAGCAGAGACCCCTGTCTCTTATACACATCTCA	STM1599-CyaA’
STM1599-CyaAR1	GGCGTAATGATTAGATTTCGCAACAAAACGCCGTTTCACTGTCTCTTATACACATCTGGT	STM1599-CyaA’
STM1633-CyaAF1	CCACAAAAAATATCTGGCATTGTCGATAGCTGGATGAAACTGTCTCTTATACACATCTCA	STM1633-CyaA’
STM1633-CyaAR1	AAGATAGCCAGCATATTACCTGATGACATAGCTTACTACTGTCTCTTATACACATCTGGT	STM1633-CyaA’
STM3595-CyaAF1	GATCTGTCCGCTGCTTACGAGATGGCGAGAAAAACGCGCCTGTCTCTTATACACATCTCA	STM3595-CyaA’
STM3595-CyaAR1	ATCCCGGCTTACGTTTTATAAGCCGGGTGACAACGTCACTGTCTCTTATACACATCTGGT	STM3595-CyaA’
PagC-CyaAF1	AAAATAAACGGCTTCAACGTCGGGGTTGGATACCGTTTCCTGTCTCTTATACACATCTCA	PagC-CyaA’
PagC-CyaAR1	TGCGGAAGGCGAACCTTCCGCATAGCTTATGCTTTTCACTGTCTCTTATACACATCTGGT	PagC-CyaA’
PagD-CyaAF1	GTCAATGACGCCAGGAAAGGGAATACTTTCTCCAGAACACTGTCTCTTATACACATCTCA	PagD-CyaA’
PagD-CyaAR1	AAGTAGTCGTTCGGCACCATTGTAGATAAATAACATTACTGTCTCTTATACACATCTGGT	PagD-CyaA’
PagK-CyaAF1	CCCTTCCCTGAAGATTGGTTTAAAATGTGTAGAGGTTATCTGTCTCTTATACACATCTCA	PagK-CyaA’
PagK-CyaAR1	TGATATGGTTACTGGCTAGTATATTAAATTTATACTCACTGTCTCTTATACACATCTGGT	PagK-CyaA’
STM2585A-CyaAF1	CCCTTCCCTGAAGATTGGTTTAAAATGTGTAGAGGTTATCTGTCTCTTATACACATCTCA	STM2585A-CyaA’
STM2585A-CyaAR1	TGATATGGTTACTGGTTAGTATATTAAATTTATACTCACTGTCTCTTATACACATCTGGT	STM2585A-CyaA’
PagJ-CyaAF1	CCCTTCCCTGAAGATTGGTTTAAAATGTGTAGAGGTTATCTGTCTCTTATACACATCTCA	PagJ-CyaA
PagJ-CyaAR1	TGATATGGTTACTGGCTAGTATATTAAATTTATACTCACTGTCTCTTATACACATCTGGT	PagJ-CyaA
SseJ-CyaAF1	AATGTTAGAAAGTTTTATAGCTCATCATTATTCCACTGAACTGTCTCTTATACACATCTCA	SseJ-CyaA’
SseJ-CyaAR1	TGTGTTTTGCTCAAGGCGTACCGCAGCCGATGGAACTTTACTGTCTCTTATACACATCTGGT	SseJ-CyaA’
SrfN-BlaRF1	TTTGAAGCCGTTGCCGAAGTTTATAACGATGCGACGAAACTGTCTCTTATACACATCTCA	SrfN-Bla
SrfN-BlaRR1	CATGCCAGTGCCCTCGTTATGGCGCAATCAGAGCTTTA CTGTCTCTTATACACATCTGGT	SrfN-Bla
PagK-BlaRF1	CCCTTCCCTGAAGATTGGTTTAAAATGTGTAGAGGTTATCTGTCTCTTATACACATCTCA	PagK-Bla
PagK-BlaRR1	TGATATGGTTACTGGCTAGTATATTAAATTTATACTCACTGTCTCTTATACACATCTGGT	PagK-BLa

STM2585A-BlaRF1	CCCTTCCCTGAAGATTGGTTTAAAATGTGTAGAGGTTATCTGTCTCTTATACACATCTCA	STM2585A-Bla
STM2585A-BlaRR1	TGATATGGTTACTGGTTAGTATATTAAATTTATACTCACTGTCTCTTATACACATCTGGT	STM2585A-BLa
PagJ-BlaRF1	CCCTTCCCTGAAGATTGGTTTAAAATGTGTAGAGGTTATCTGTCTCTTATACACATCTCA	PagJ-Bla
PagJ-BlaRR1	TGATATGGTTACTGGCTAGTATATTAAATTTATACTCACTGTCTCTTATACACATCTGGT	PagJ-BLa
STM0082-RF1	GCGCAAACCTTATCATCAATGCAACAGGAACAGAACTATGATTCCGGGGATCCGTCGACC	ΔSTM0082
STM0082-RR1	TGGCGCAATCAGAGCTTTATTTCGTCGCATCGTTATAAACGTGTAGGCTGGAGCTGCTCC	ΔSTM0082
PagK-RF1	TAAGGATAGTGTGCCACAATTTAACAGGTAACATATTATGATTCCGGGGATCCGTCGACC	Δ*pagK*
PagK-RR1	TATATTAAATTTATACTCAATAACCTCTACACATTTTAAAGTGTAGGCTGGAGCTGCTCC	Δ*pagK*
STM2585A-RF1	TATCTATAGTAAAGGATGCTGTAACCGTAAGGATAGTGTGATTCCGGGGATCCGTCGACC	ΔSTM2585A
STM2585A-RR1	TATATTAAATTTATACTCAATAACCTCTACACATTTTAAAGTGTAGGCTGGAGCTGCTCC	ΔSTM2585A
PagJ-RF1	TAAGGATAGTGTGCCACAATTTAACAGGTAACATATTATGATTCCGGGGATCCGTCGACC	Δ*pagJ*
PagJ-RR1	ATATATAAATTTATACTCAATAACCTCTACACATTTTAAAGTGTAGGCTGGAGCTGCTCC	Δ*pagJ*
ssaK-RF1	CTCTTACCGAGATTATCTTCATGGAGCATTTGCTATGAGTGTGTAGGCTGGAGCTGCTTC	Δ*ssaK*
ssaK-RF2	TTAATATTCATCGCTACCTCTTTTATCTTCACCATTACGTATATGAATATCCTCCTTAGT	Δ*ssaK*
invA-RF1	AAAGCTGTCTTAATTTAATATTAACAGGATACCTATAGTG GTGTAGGCTGGAGCTGCTTC	Δ*invA*
invA-RR1	TTCCTTAATTAAGCCCTTATATTGTTTTTATAACATTCAC ATATGAATATCCTCCTTAGT	Δ*invA*
flgB-RF1	AAGCTGTCGGCTGAATTTTGCCATTTGCGGAGGAGATATG GTGTAGGCTGGAGCTGCTTC	Δ*flgB*
flgB-RR1	TTAACAGCGCCACGAATTAGTTTCCTCCCTGTAGCACATT ATATGAATATCCTCCTTAGT	Δ*flgB*
STM0082-CF1	TATGAATTCCTACGCGTAAGATCCGTCTCTTC	pSrfN
STM0082-CR1	TTATTCTAGAATCAGAGCTTTATTTCGTCGCATC	pSrfN
STM2585A-CF1	AAAGAATTCATAAAACGCGTGACAAAAACCATG	pPagK2
STM2585A-CR1	AAATCTAGACCTGTCTGTCATAACTGATATGG	pPagK2
