# Supplementary material for: Systems analysis of multiple regulator perturbations allows discovery of virulence factors in Salmonella
Source: BMC Syst Biol. 2011 Jun 28;5:100. doi: 10.1186/1752-0509-5-100 (PMC3213010; doi:10.1186/1752-0509-5-100)
Supplement: Additional file 14 — Table S5. Primers used in qRT-PCR. [file 1752-0509-5-100-S14.PDF]

Additional file 14

Supplementary Table S5. Primers used in qRT-PCR

Name	Forward primers (5' to 3')	Reverse primers (5' to 3')
gyrB	GGCTACAGCAAAAAAGCCAAA	GGAGAATTTCGGATCCGGTACT
srfN	AAAAAACGCATTATTGCCGCCG	TTGAAGTGGCTGATTTCTTGTTTAG
pagC	ACAGATGAATCCGCTGGAGAA	CCGACGTTGAAGCCGTTTATT
pagD	TATTCCCTTTCCTGGCGTCAT	AAAAGCAAGCCAGGCAGATTAG
pagK1/pagK2/pagJ	CACGTCAAGAGCGTATTTTTAGCA	ATGATCTTGAGAGTCTGCCGCTAT
pagK1/pagK2/pagJ	TCTTACAATAGCGGCAGACTCTCA	CAGGCCAAAGATTACACCACTTT
ssaE	CCGCAGCAATATCAGCAAAA	AAGTGCGCTGTTATGGTAACGA
sseA	AAAGGCTGCGTTTAGTGAATATCG	TGACTCACCTTAGCCCGGATT
sscA	GGCTCGCTGCGTATGTTGTT	GCCGGCGAATTCTTTTACCT
ssaG	ATGATTTGCTCAACCCAGAA	TTTAGCAATGATTCCACTAAGCA
ssaH	TTCCCAGGTACATGCGATGTTA	TCATTTAAACCCGCCAACAATA
ssaN	GATGCAACGTCTGAGGCTGAA	GGCAACCACGCATTTAACAA
srfH	GATACCCCCCCTGAAATGAGTT	GTGACAAATCGTCCAGATGCA
sseJ	GGAAGCTTTTGGTCTTGCAAA	CTGTCGCCAAAAAATACCAGTCT
phoP	TCCGGATATCGCTATTGTCGAT	ACCAGAACCGGCAGTGAAAC
ssrB	CCGCAGGTGCTAATGGCTAT	TTGGGTCAATGTAACGCTTGTT
